# Understanding Polycaprolactone Degradation: Molecular Mechanisms and Implications for Biomedical Device Design

**DOI:** 10.3390/ma19183894

**Published:** 2026-09-12

**Authors:** Paulina Dziemiańczyk, Dawid Łysik, Francois Vernay, Joanna Mystkowska

**Affiliations:** 1 Institute of Biomedical Engineering, Faculty of Mechanical Engineering, Bialystok University of Technology, Wiejska 45C, 15-351 Bialystok, Polandj.mystkowska@pb.edu.pl (J.M.); 2 Laboratoire PROMES CNRS (UPR-8521), Université de Perpignan Via Domitia, Tecnosud, 66100 Perpignan, France; francois.vernay@univ-perp.fr

**Keywords:** polycaprolactone, biodegradation, degradation mechanisms, degradation kinetics, molecular weight, biomedical devices, tissue engineering

## Abstract

Polycaprolactone (PCL) is a widely used biodegradable polyester in tissue engineering, drug delivery, and temporary implant design. While its favorable processability, biocompatibility, and low melting temperature are highly advantageous, its slow and condition-dependent degradation remains a major limitation for precise temporal control in biomedical applications. Despite extensive literature on PCL, a critical knowledge gap remains in linking fundamental molecular chain scission directly to macroscopic structural evolution, mechanical failure, and predictable in vivo device performance. To address this, this review provides a comprehensive synthesis of PCL degradation mechanisms, with a particular emphasis on PCL-bioceramic composites designed for hard tissue engineering. We elucidate the progressive degradation pathway—distinguishing between initial hydrolytic chain scission, oligomer formation, the generation of low-molecular-weight degradation products, and their subsequent metabolic fate under physiological conditions. Furthermore, this review critically evaluates how fundamental variables—specifically molecular weight, crystallinity, bioceramic fillers, device geometry, and physiological environments—alter degradation kinetics. By connecting molecular weight reduction to subsequent mass loss, thermal behavior, and mechanical deterioration, we establish a framework for understanding how structural reorganization and crystallinity evolution govern material failure. This review bridges the gap between simplified in vitro models and complex in vivo realities, supporting the rational design of composite biomedical devices with tailored, predictable resorption profiles.

## 1. Introduction

### 1.1. Theoretical Background

Among biodegradable polymers, aliphatic polyesters play a significant role. This group includes widely used materials such as poly(lactic acid) (PLA), poly(glycolic acid) (PGA), poly(lactic-co-glycolic acid) (PLGA), polyhydroxyalkanoates (PHAs), and poly(ε-caprolactone) (PCL) [1]. The degradation behavior and biofunctional properties of PLA, PGA, PLGA, and PHAs have been extensively investigated [2,3,4]. In contrast, polycaprolactone has received comparatively less attention, despite its potential in long-term biomedical applications.

PCL is a biocompatible, biodegradable, and bioresorbable aliphatic polyester. While it shares the broader synthetic polyester classification with PLA and PGA [5], PCL is distinct as a poly(ε-hydroxy acid) rather than an α-hydroxy derivative. Polycaprolactone (PCL), first synthesized in the early 1930s via the ring-opening polymerization of the cyclic ε-caprolactone monomer [6], comprises repeating (C_6_H_10_O_2_)_n_ units. The degree of polymerization (n), and consequently the macromolecular weight, directly dictates the kinetics of ester-bond hydrolysis, thereby determining the polymer’s persistence in biological environments. Structurally, PCL is characterized by a low glass transition temperature (T_g_) of approximately −60 °C and a melting point (T_m_) within the range of 56–64 °C; notably, both these thermal traits and its high intrinsic crystallinity are dictated by the conformational flexibility of its long aliphatic -(CH_2_)_5_- repeating units.

However, due to this hydrophobic and semi-crystalline nature, PCL exhibits a protracted degradation rate, typically spanning 2 to 4 years [7]. This slow mass loss is primarily governed by these same non-polar methylene segments, which drastically restrict water diffusion while reducing the steric density of hydrolytically susceptible ester bonds relative to shorter-chain polyesters like PGA or PLA. Furthermore, the tightly packed crystalline domains present significant steric barriers that highly restrict moisture penetration, localizing initial hydrolytic cleavage predominantly to the more accessible amorphous phases.

The extent of autocatalytic acceleration in PCL degradation is strongly dependent on device geometry and porosity. In highly porous scaffolds or thin films, PCL generally lacks the pronounced bulk autocatalysis characteristic of PLA or PLGA. Because its byproduct, 6-hydroxyhexanoic acid (6-HCA), is generated slowly, it undergoes outward diffusion and dilution into the surrounding milieu. Conversely, in massive, non-porous geometries (e.g., solid composite blocks for bone defect regeneration), the extended diffusion path hinders the escape of 6-HCA, which can lead to acidic accumulation in the core and a subsequent acceleration of internal matrix erosion. The selected properties of PCL are summarized in Table 1.

In tissue engineering, controlled drug release from scaffolds enables sustained, localized therapeutic effects while minimizing systemic side effects [10]. However, achieving a stable and well-regulated release profile without compromising the scaffold’s structural integrity remains a significant challenge [11]. In this context, poly(ε-caprolactone), due to its slow degradation rate and favorable physicochemical properties, represents a promising material for such applications. Moreover, PCL is non-toxic and has demonstrated cytocompatibility with several types of biological tissues, further supporting its suitability for tissue engineering [12]. PCL is also FDA-approved for specific biomedical devices and drug-delivery systems, which makes an accurate understanding of its degradation behavior a clinical, and not merely academic, requirement. Additionally, PCL exhibits advantageous and tunable mechanical properties—such as Young’s modulus, elasticity, tensile strength, and elongation at break—which enable its use in a wide range of biomedical and paramedical applications, including wound dressings, contraceptive devices, and dental materials [13].

Compared with other commonly used biodegradable polyesters, PCL exhibits favorable viscoelastic properties that facilitate melt processing, shaping, and handling. As a result, it can be fabricated into a wide variety of structures, including microspheres, fibers, micelles, films, nanofibers, and foams [14,15,16]. Furthermore, PCL can be readily blended or copolymerized with other polymers and bioceramics, enabling the development of materials with tailored physicochemical properties and controlled biodegradability [17].

Despite the extensive body of literature on general PCL degradation, predicting its long-term behavior remains challenging. A critical appraisal of current literature reveals frequent methodological weaknesses and contradictions—such as the interchangeable use of the terms ‘degradation’ and ‘erosion’, varying in vitro testing environments that fail to accurately mimic dynamic in vivo conditions, and conflicting reports regarding the exact role of polymer crystallinity. Previous reviews often treat degradation sequentially, leaving a critical knowledge gap regarding the simultaneous interplay between molecular-level chain scission, structural evolution (e.g., chemicrystallization), and the resulting macroscopic mechanical failure. Moreover, as modern applications, particularly in bone tissue engineering, increasingly rely on the incorporation of bioceramic additives or active ion dopants to enhance bioactivity, there is a pressing need to systematically evaluate how these inorganic phases alter fundamental hydrolytic pathways. Therefore, this review aims to bridge this gap by synthesizing current knowledge on PCL degradation, with a dedicated emphasis on PCL-bioceramic composites. By elucidating the connections between molecular mechanisms and structural property changes, this analysis advances beyond previous literature to provide a foundational framework for designing composite devices with predictable clinical performance.

### 1.2. Literature Search Methodology

**Figure 1:**
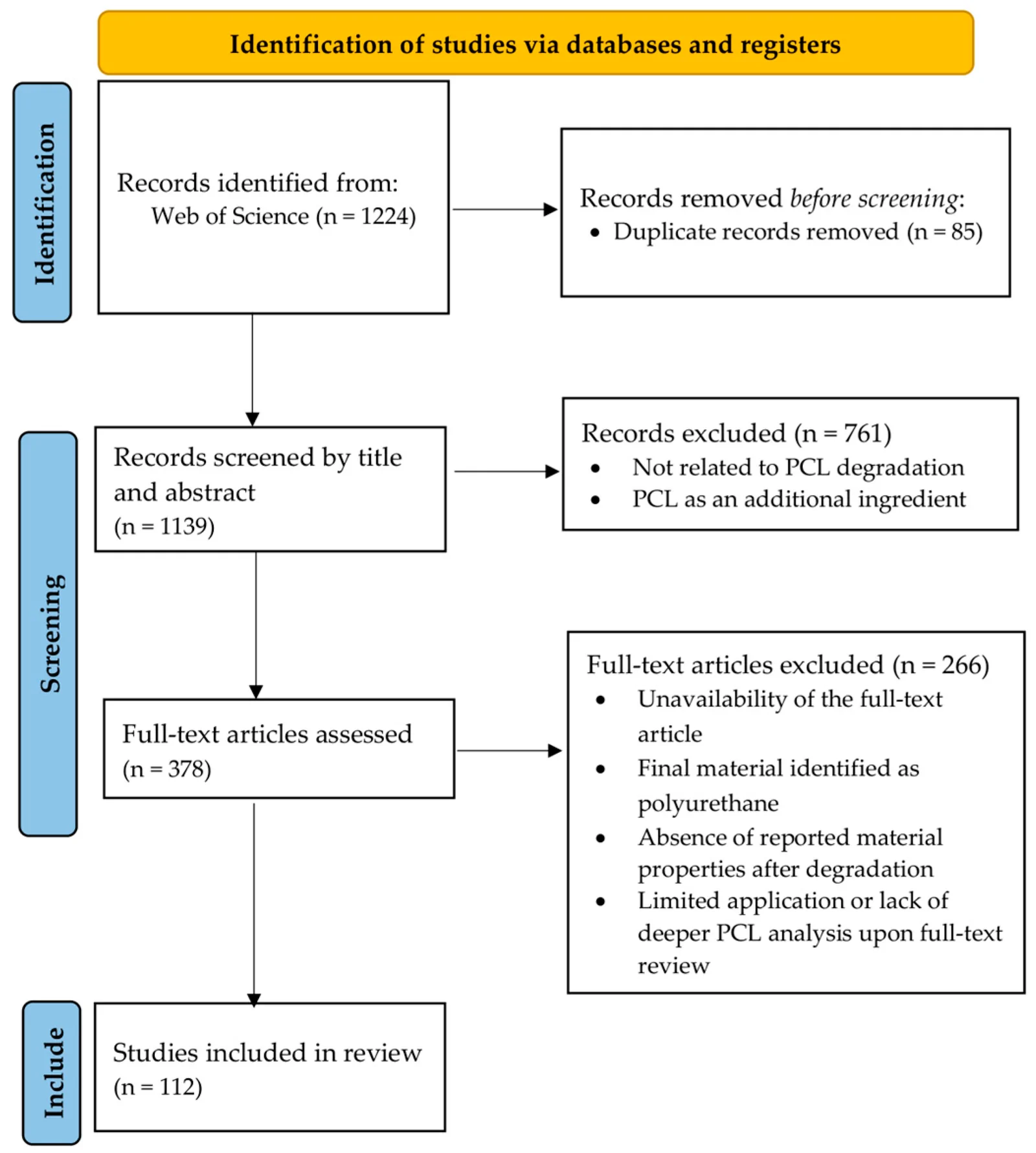
The flowchart of the literature selection process illustrates the identification, screening, and inclusion of sources analyzed in this study.

A literature search was conducted using the Web of Science database. The aim of this review is to systematically present literature reports on the degradation of polycaprolactone-based materials. We focused on how degradation affects material properties, especially molecular weight, mass loss, and mechanical and thermal properties. We also considered the influence of material geometry and incubation environment on the final properties of the material. An overview of the literature identification and selection process is provided in Figure 1.

The objective of this work was to summarize the current knowledge on the degradation of polycaprolactone materials, with particular emphasis on bioceramic additives. A comprehensive literature search of the Web of Science database was conducted for articles published between January 1992 and June 2026. A search of the Web of Science database was conducted using the terms “polycaprolactone” or “poly(ε-caprolactone),” and “degradation” or “degradability”, and “molecular weight”, applied simultaneously to all fields (title, abstract, and keywords). The inclusion of “molecular weight” as a mandatory search term was intentional; as this review focuses on the fundamental mechanisms of hydrolytic cleavage, studies reporting molecular weight reduction were prioritized over those reporting solely superficial mass loss or macroscopic erosion.

During the screening and full-text assessment phases, strict eligibility criteria were applied to minimize subjectivity. The inclusion criteria were defined as follows: (1) PCL served as the primary polymeric matrix of the investigated material; (2) strictly controlled degradation tests were performed; (3) quantitative evaluation of the material’s structural, mechanical, or thermal properties post-degradation was reported; and (4) studies investigating pure PCL or PCL/bioceramic composites were specifically targeted to evaluate the influence of inorganic phases on degradation kinetics.

The exclusion criteria comprised: (1) unavailability of the full-text article; (2) systems where PCL was not the main structural component (e.g., constituting less than 50 wt% or used solely as a coating); (3) studies where the final synthesized material was a crosslinked polyurethane rather than a linear PCL-based system; and (4) investigations lacking comprehensive polymer analysis post-degradation (e.g., reporting only overall construct mass loss without characterizing changes in the polymer’s molecular weight, crystallinity, or thermal behavior).

### 1.3. Mathematical Models and Formulation

As part of a comprehensive literature review on the degradation of polycaprolactone, this study analyzed changes in key polymer properties over time. The primary focus was placed on three critical areas: molecular weight, mechanical performance, and thermal characteristics.

To allow for a direct comparison across different studies and datasets, a normalization procedure was applied to the collected data. Each measured property value was divided by its corresponding initial value, as shown in the following equation:

(1) $$P_{\mathrm{norm}}(t)=\frac{P_{t}}{P_{0}}$$

where P_norm_(t) is the normalized property value at degradation time *t*, *P_t_* the measured value at time *t*, and *P*_0_ is the initial value measured before degradation. Accordingly, the initial normalized value was 1, which provided a common reference point for assessing the relative retention or loss of individual properties during degradation.

The time-dependent decrease in the number-average and weight-average molecular weights was described using an apparent first-order exponential decay model, consistent with previous kinetic analyses of hydrolytically degrading polycaprolactone systems [18,19]. This model was applied separately to both the number–average molecular weight (M_n_), and the weight–average molecular weight (M_w_) (2):

(2) $$M_{i(t)}=M_{i}\left(0\right)e^{-\lambda^{\prime }t},i=n,w$$

where M_i_(t) is the molecular weight after degradation time t, M_i_(0) is the corresponding initial molecular weight, and *λ*’ is the apparent first-order molecular-weight decay constant. Separate values of $\lambda_{n}^{\prime }$ and $\lambda_{w}^{\prime }$ were determined because M_n_ and M_w_ may respond differently to polymer-chain scission and changes in the molecular-weight distribution. The degradation time was expressed in days; therefore, *λ*’ was expressed in day^−1^. A higher value of $\lambda_{i}^{\prime }$ indicates a faster reduction in molecular weight.

To effectively visualize the degradation kinetics over the broadly varying time scales reported in the literature, a logarithmic scale was applied to the time axis. The apparent induction period observed in the initial phase of the normalized M_n_ and M_w_ decay curves is an inherent visual effect of projecting the first-order exponential model onto a semi-logarithmic plot, rather than indicating an explicit time-delay parameter.

For the remaining properties, including mechanical and thermal parameters, classical linear regression was applied to the normalized data (3):

(3) *P_norm_*(*t*) = *at* + *b*

where P_norm_(t) is the normalized value of the analyzed property at degradation time *t*, *a* is the slope representing the average change in the normalized property per unit of time, and *b* is the intercept of the fitted regression line. Negative values of *a* index indicate a decrease in the analyzed property over time, while positive values indicate an increase.

Unlike molecular-weight reduction, for which exponential models have previously been used to describe PCL degradation, no established mathematical model was identified in the reviewed literature for describing the time-dependent evolution of the mechanical and thermal properties analyzed in the present study. Therefore, linear regression was used as a descriptive approach to quantify the overall direction and approximate magnitude of the observed trends rather than as a kinetic or mechanistic model of degradation. In particular, the slope *a* was used to determine whether the analyzed property exhibited an overall increasing or decreasing tendency over the investigated degradation period.

For properties exhibiting complex, non-linear, or biphasic behavior, the linear regression was applied across the entire degradation period. In such cases, the fitted slope should be interpreted only as a summary descriptor of the overall trend and should not be taken to imply that the property changes linearly throughout the degradation process. The coefficient of determination (*R*^2^) for the corresponding linear fits is provided in the relevant figures in Section 3.

To prevent potential artifacts arising from inter-study heterogeneity, each experimental curve and its corresponding mathematical fit were derived exclusively from an individual dataset within a single study. Regardless of the model applied, data from different literature sources were not pooled or aggregated during the fitting process, thereby preserving the specific experimental conditions (e.g., sample geometry, degradation medium, and initial polymer characteristics) of each original investigation (the fitted parameters are provided in the Supplementary Materials).

### 1.4. Hydrolytic Degradation

PCL is a semi-crystalline, hydrophobic, aliphatic polyester that degrades primarily through the random hydrolytic cleavage of its ester linkages [20,21]. In aqueous environments, water molecules attack the ester bonds along the polymer backbone. This scission event results in the formation of two distinct terminal groups: a hydroxyl group and a carboxyl group [20].

The hydrolytic degradation of PCL is often considered autocatalytic under specific conditions. Carboxyl end groups generated during the initial hydrolytic cleavage of ester bonds increase the local acidity within the polymer matrix. As these acidic degradation products accumulate, they further promote ester hydrolysis, thereby accelerating chain scission and molecular weight reduction [20,22,23]. However, the extent of this autocatalysis is highly dependent on device geometry, porosity, and the surrounding degradation environment, as thin or highly porous structures allow acidic byproducts to diffuse away before significant internal accumulation occurs.

PCL degradation exhibits a biphasic profile. The first stage is characterized by a progressive decrease in number-average molecular weight (M_n_) and weight-average molecular weight (M_w_) caused by random chain scission, while mass loss remains negligible. In the second stage, once the molecular weight falls below a critical threshold, typically reported to be in the range of 3000–5000 g/mol for certain specific systems, rather than representing a universal value, the resulting oligomers become sufficiently small and water-soluble to diffuse from the matrix. This transition leads to measurable mass loss, structural disintegration, and eventual clearance of the material [24,25]; however, it is important to note that the physical removal of degradation products does not necessarily imply immediate physiological reabsorption.

**Figure 2:**
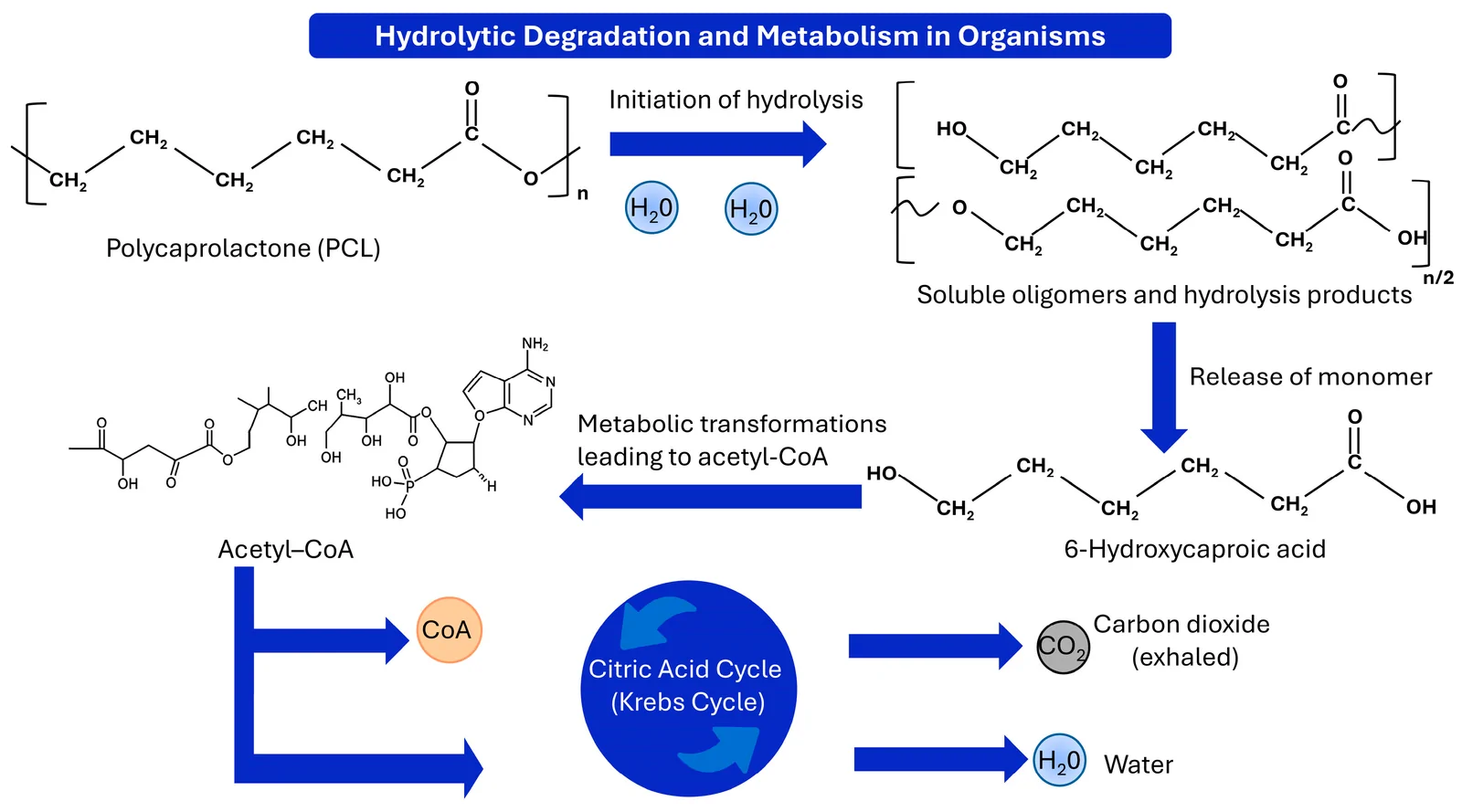
The in vivo bioresorption of polycaprolactone initiates with non-enzymatic ester hydrolysis, where water molecules cleave the polymer backbone to generate soluble oligomers and low molecular weight products. Continuous random chain scission causes the entire matrix to disintegrate, leading to the release of the constituent 6-hydroxycaproic acid monomers. These degradation fragments are subsequently internalized via cellular pathways, where they undergo sequential metabolic transformations to yield acetyl coenzyme A (acetyl-CoA). This metabolic intermediate directly enters the citric acid (Krebs) cycle to be fully broken down by the host organism. The polymer structure is entirely mineralized and eliminated from the body as non-toxic, physiological end-products, specifically exhaled carbon dioxide and water [13,33].

The rate of hydrolysis is governed by the accessibility of water to the ester bonds. Due to the hydrophobic nature of PCL and its semi–crystalline structure, water uptake is relatively slow compared to more hydrophilic polyesters [20]. As illustrated in Figure 2, water molecules penetrate the polymer matrix and preferentially diffuse into the amorphous regions where the chains are more loosely packed and therefore more susceptible to hydrolytic attack. Water molecules diffuse preferentially into the amorphous regions of the polymer matrix, where the chains are loosely packed [20,21].

Consequently, hydrolytic degradation initiates in these amorphous domains, while the densely packed crystalline lamellae initially resist water penetration. As the amorphous chains are cleaved, the remaining chain segments gain mobility and often realign into new crystalline structures. This phenomenon, known as chemicrystallization, leads to a measurable increase in crystallinity during the early to intermediate stages of degradation. Only after the degradation of the amorphous regions is the hydrolytic attack effectively transferred to the crystalline domains [20]. This relationship—where the continuous decrease in molecular weight (degradation) drives changes in crystallinity and the deterioration of mechanical properties—precedes the subsequent mass loss (erosion) that governs PCL’s behavior in biomedical applications.

The kinetics and mechanism of PCL degradation are heavily influenced by the pH and temperature of the surrounding medium.

The degradation mechanism shifts significantly depending on the pH. Under acidic conditions (e.g., HCl exposure), protons (H^+^) are small enough to penetrate the polymer bulk rapidly. This catalyzes random chain scission throughout the matrix, resulting in “bulk degradation” characterized by a uniform decrease in molecular weight and mechanical embrittlement, often with limited immediate mass loss. Because it reproduces the bulk-degradation mode and the acidic microenvironment that autocatalysis creates inside the matrix in vivo, the acidic model is often considered by some authors as a useful representation of internal core conditions, although it does not fully replicate the complexity of physiological environments. Conversely, in alkaline environments (e.g., NaOH), the degradation is driven by hydroxyl ions (OH^−^). This reaction is rapid and largely confined to the polymer-water interface, because hydroxyl ions react faster than they can diffuse into the bulk (reaction rate exceeds diffusion rate). This results in “surface erosion,” where mass is lost from the exterior while the internal molecular weight remains relatively stable [20], though this effect can vary depending on medium concentration and polymer porosity.

Temperature strongly affects the hydrolysis rate. Degradation rates follow an Arrhenius relationship, increasing at elevated temperatures [26]. For instance, the half-life of PCL molecular weight was found to be approximately 52 weeks at 37 °C but dropped to 13 weeks at 55 °C [27]. Higher temperatures raise the intrinsic rate constant of ester hydrolysis (the Arrhenius term) and increase the diffusivity of water in the matrix, both of which speed degradation [26,28].

Understanding the distinction between degradation (defined strictly as the process of polymer chain scission) and erosion (defined as macroscopic material mass loss) is critical for predicting the lifetime and mechanical performance of PCL implants. In physiological conditions (neutral pH, 37 °C), PCL typically undergoes bulk degradation that eventually leads to bulk erosion [20,26,29,30]. This regime occurs when the rate of water diffusion (D) into the polymer matrix is faster than the rate (R) of the hydrolysis reaction (D_water_ > R_reaction_), a balance that depends heavily on variables such as temperature, crystallinity, initial molecular weight, medium concentration, and device geometry. Water penetrates the entire volume of the device before significant degradation occurs. As a result, the molecular weight decreases uniformly throughout the implant, and mechanical properties (such as tensile strength) deteriorate significantly before any mass loss or dimensional changes are observed [21,26,31].

Conversely, surface erosion occurs when the rate of the hydrolysis reaction is faster than the rate of water diffusion (R_reaction_ > D_water_) [20,28]. This is often observed in PCL under accelerated alkaline conditions or enzymatic attack (e.g., by lipases). In this scenario, the material degrades from the outside, maintaining its bulk molecular weight and structural integrity in the core while the device dimensions shrink over time. This mechanism is desirable for drug delivery applications where zero-order release kinetics are required, though unmodified PCL rarely exhibits this behavior in vivo [22,32].

The degradation of PCL is a complex interplay between molecular weight, crystallinity, and environmental conditions. While the fundamental chemical mechanism remains ester hydrolysis, the macroscopic behavior ranges from bulk erosion in physiological fluids to surface erosion in alkaline or enzymatic environments. Biomedical Engineers must account for these variables, particularly the lag time between molecular weight reduction and mass loss characteristic of bulk erosion, to ensure the mechanical integrity of PCL scaffolds matches the rate of tissue regeneration.

### 1.5. Enzymatic Degradation

**Figure 3:**
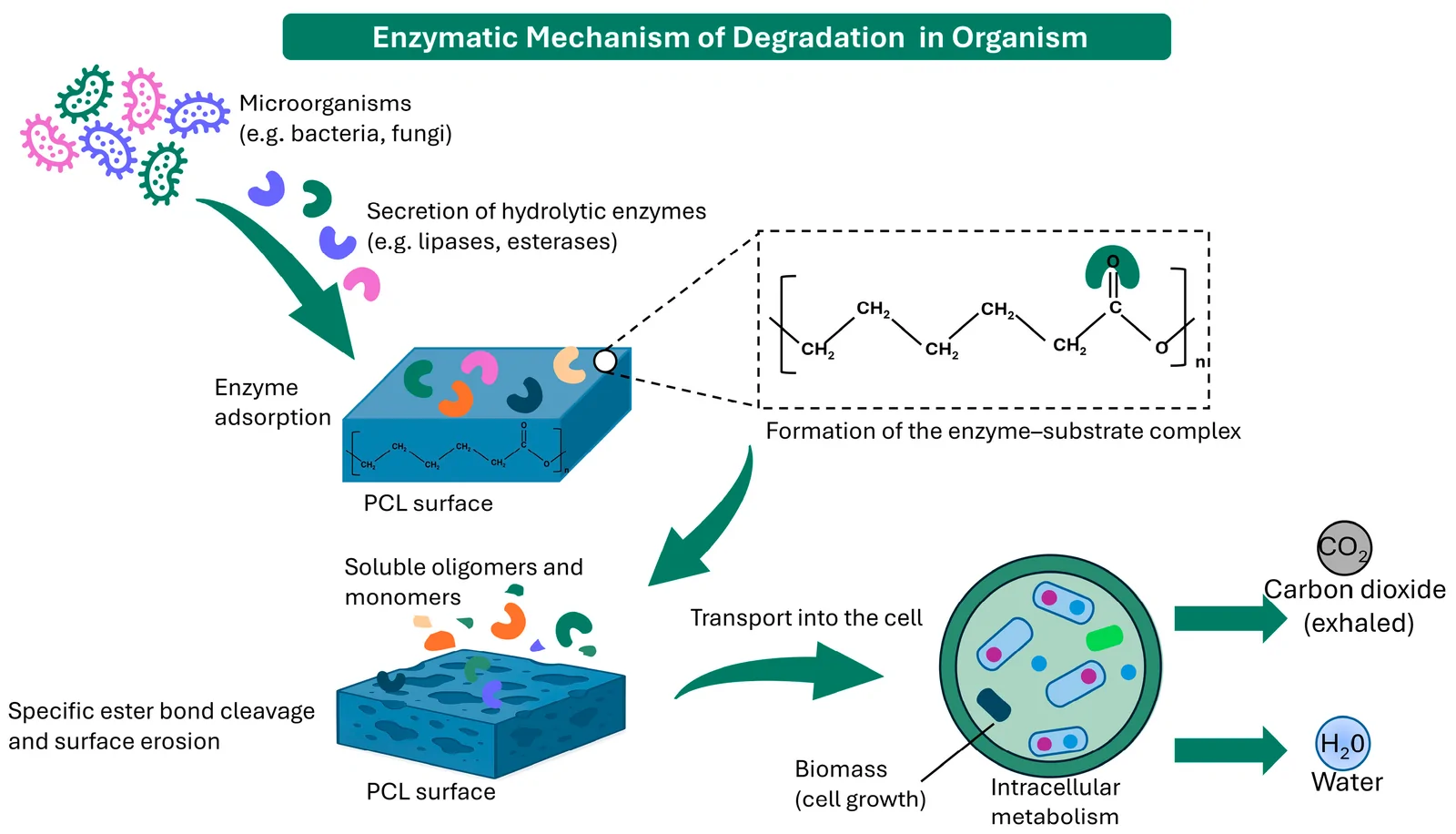
Microorganisms (bacteria and fungi) secrete extracellular lipases and esterases that adsorb onto the PCL surface. The enzymes form an enzyme–substrate complex by targeting the carbonyl carbon (C=O) within the polymer’s ester bonds. This specific cleavage drives surface erosion, breaking down the matrix into soluble oligomers and monomers. These fragments are transported into the cell, where they enter intracellular metabolic pathways to support biomass growth. Finally, the polymer is fully mineralized into non-toxic end-products, carbon dioxide, and water [33,42,55].

The ester bonds that constitute the PCL backbone are susceptible to hydrolysis by several classes of enzymes, most of which belong to the broader superfamily of serine hydrolases and share a conserved catalytic triad consisting of serine, histidine, and aspartate (or glutamate) residues [34,35]. As illustrated in Figure 3, extracellular enzymes secreted by microorganisms adsorb onto the PCL surface and catalyze the cleavage of ester bonds, initiating polymer chain scission and subsequent erosion. The most studied classes of enzymes acting on PCL include the following processes. Lipases are interfacially activated esterases that act preferentially at the water–lipid interface. Most possess a hydrophobic lid domain that undergoes conformational rearrangement upon contact with a hydrophobic substrate surface, thereby exposing the catalytic site [35]. *Candida antarctica* lipase B (CALB) represents a notable exception, as its active site is not covered by a conventional mobile lid and the enzyme exhibits little or no classical interfacial activation. Among the lipases most employed in PCL degradation studies are those from *Pseudomonas* spp., CALB, *Rhizopus delemar*, *Mucor miehei*, and *Thermomyces lanuginosus*. Bacterial lipases, particularly those derived from *Pseudomonas* spp., are frequently reported to exhibit high activity toward PCL [34,36,37]. Lipase isolated from *Lactobacillus plantarum* has also been shown to promote substantial PCL degradation, particularly when embedded directly within the polymer matrix [38].

Cutinases are serine esterases originally associated with the degradation of cutin, the structural lipid polymer of the plant cuticle. Unlike lipases, cutinases lack a hydrophobic lid and do not require interfacial activation, allowing them to act on both soluble and insoluble substrates [35,39]. Murphy et al. [39] demonstrated that the PCL depolymerase secreted by *Fusarium solani* is a cutinase, establishing a direct mechanistic link between the enzymatic degradation of plant cutin and synthetic polyester biodegradation. This conclusion was supported by the inability of a cutinase-deficient mutant to degrade PCL, in contrast to the corresponding wild-type strain, as well as by the induction of cutinase activity by PCL hydrolysis products. More recently, Shi et al. [36] directly demonstrated the hydrolysis of PCL films by *F. solani* cutinase, which produced progressive, layer-by-layer surface erosion and an approximately 80.8% mass loss after 72 h. Cutinase-associated PCL degradation has also been reported in bacteria. *Bacillus* sp. KY0701 was shown to degrade PCL films and produce a thermostable cutinase, whereas a *Pseudomonas pachastrellae*-related strain, TKCM 64, produced a PCL hydrolase whose induction profile was consistent with cutinase-like activity [40,41].

Carboxylesterases act on smaller, more water-soluble substrates than lipases and are not interfacially activated. Nonetheless, certain esterases—particularly the thermophilic esterase from the archaeon *Archaeoglobus fulgidus*—have demonstrated catalytic activity toward PCL and other aliphatic polyesters [35,42]. Their role is thought to be particularly relevant in the later stages of degradation, when low-molecular-weight oligomers and water-soluble fragments become available as substrates.

Although proteinase K is a serine endopeptidase rather than an esterase, it has been reported to exhibit moderate activity on certain aliphatic polyesters, particularly poly(L-lactic acid). Its activity on PCL is limited relative to lipases and cutinases, but it has been employed as a comparative benchmark in degradation studies [34,43].

The catalytic mechanism by which serine hydrolases cleave PCL ester bonds follows the classical two-step acyl-enzyme intermediate pathway [35,43]. In the first step (acylation), the hydroxyl group of the catalytic serine residue performs a nucleophilic attack on the carbonyl carbon of the ester bond, forming a tetrahedral intermediate that is stabilized by the oxyanion hole of the enzyme. The collapse of this intermediate results in the release of the alcohol-leaving group (ω-hydroxyl fragment of the PCL chain) and the formation of an intermediate in the form of an acyl-enzyme. In the second step (deacylation), a water molecule (activated by the catalytic histidine) attacks the acyl-enzyme intermediate, regenerating the free enzyme and releasing a carboxylic acid-terminated PCL fragment or 6-HCA monomer [35,44].

The specificity and efficiency of this reaction depend critically on the ability of the enzyme to adsorb to the polymer surface, the accessibility of ester bonds within the semi-crystalline matrix, and the local chain mobility at the point of attack. Molecular dynamics simulations and quantum mechanics/molecular mechanics studies have provided atomic-level insight into substrate binding and transition-state geometries, revealing that the flexibility of the PCL chain in the amorphous phase facilitates productive orientation within the enzyme active site [35].

Enzymes acting on PCL can be classified according to their mode of chain scission. Endo-type (random) scission involves cleavage of ester bonds at arbitrary positions along the polymer backbone, generating a distribution of oligomeric products. Exo-type (chain-end) scission involves sequential removal of monomer units (6-HCA or ε-caprolactone) from chain termini [34,44]. Rosato et al. [34] showed that the dominant cleavage mode is enzyme dependent. Lipases from *Pseudomonas fluorescens*, *Pseudomonas* sp. and *Alcaligenes* sp. acted through endo-type scission, as indicated by the accumulation of oligomeric products, whereas CALB, lipase B from *Candida antarctica* and cutinase displayed a stronger exo-type contribution and produced a higher proportion of 6-HCA. In a non-aqueous system, Aris et al. [44] reported that CALB-mediated degradation of PCL in toluene involved an initial random-scission phase followed by chain-end cleavage. Cyclic dicaprolactone, tricaprolactone and tetracaprolactone were also detected and attributed to intramolecular transesterification through a back-biting mechanism rather than to hydrolytic endo-type scission.

Because enzymatic degradation is predominantly a surface-mediated process, the ratio of surface area to volume of a PCL device is a critical determinant of its degradation rate. Electrospun nanofibrous scaffolds, with their extremely high specific surface area, undergo enzymatic degradation faster than bulk films or 3D-printed constructs of equivalent mass [45,46]. Conversely, dense, thick-walled implants present a limited surface for enzyme adsorption and exhibit correspondingly slower degradation kinetics. Device porosity, pore interconnectivity, and surface roughness further modulate the accessibility of the polymer surface to enzymes in the surrounding medium [33,47].

The distinction between surface erosion and bulk degradation is of central importance in understanding the behavior of PCL under enzymatic attack. In the absence of enzymes, PCL degrades predominantly through bulk hydrolysis: water penetrates the entire polymer matrix, and chain scission occurs homogeneously throughout the sample volume, resulting in progressive molecular weight loss without significant mass change until the chains are short enough to dissolve [19,33]. Enzymatic degradation, by contrast, typically proceeds via surface erosion, because the macromolecular size of enzymes (typically 30–60 kDa) is widely proposed to prevent their diffusion into the polymer bulk [34,48,49].

Rosato et al. [34] provided direct evidence for the surface erosion mechanism by showing that the molecular weight and thermal stability of residual PCL solids remained essentially unchanged even after substantial mass loss during enzymatic degradation—a finding inconsistent with bulk hydrolysis, which would produce a decrease in M_n_ throughout the sample. Hayashi et al. [48] confirmed surface-limited degradation using scanning electron microscopy (SEM), which revealed progressive erosion from the fiber surface toward the core with no evidence of internal pore formation or internal chain scission. PCL films implanted in the rat intestine—where pancreatic lipase concentration is high—degraded via surface erosion, whereas films implanted in the stomach (where lipase activity is lower and acidic hydrolysis predominates) followed a bulk erosion pathway [50].

The practical consequence of surface erosion for device design is that device geometry becomes the primary determinant of degradation rate, mechanical integrity is maintained for a longer proportion of the device lifetime (because the load-bearing cross-section is reduced gradually from the outside), and degradation products are released at a more constant rate than in bulk-degrading systems. These features are particularly advantageous for controlled drug delivery applications [13].

In the physiological environment, PCL degradation is dominated by slow, non-enzymatic bulk hydrolysis. Enzymatic contributions may nevertheless become locally relevant at the cell–material interface and during the later clearance of low-molecular-weight fragments. As part of the foreign body response, monocyte-derived macrophages and multinucleated foreign-body giant cells (FBGCs) accumulate at the implant surface [51,52]. These cells release hydrolytic enzymes, including esterases, lipases, and acid phosphatases, into the confined space between the cell membrane and the polymer, creating a locally concentrated enzymatic microenvironment that can complement passive hydrolysis and facilitate surface degradation and fragment removal [52].

The in vivo degradation of PCL is further complicated by the phenomenon of phagocytosis of small polymer fragments. As surface erosion generates microparticulate debris, macrophages and FBGCs internalize these fragments and subject them to intracellular enzymatic degradation in the phagolysosomal compartment, where the acidic pH (approximately 4.5–5.0) and high local enzyme concentration significantly accelerate hydrolysis [52,53]. The ultimate degradation product, 6-hydroxyhexanoic acid, is proposed to enter the citric acid cycle and be metabolized to carbon dioxide and water, rendering the process generally considered biologically benign [13,54].

A key challenge in the design of PCL-based biomedical devices is matching the degradation timeline to the functional requirements of the application. For drug delivery implants, the device must maintain structural integrity until the drug payload is exhausted, after which rapid resorption is desirable. For tissue engineering scaffolds, the rate of scaffold degradation must be coordinated with the rate of tissue regeneration to ensure continuous mechanical support [13].

## 2. Factors Governing Degradation Rate

In vivo, PCL typically degrades over two to four years, depending on initial molecular weight, specimen geometry, and the local environment. The initial molecular weight (M_n_ ≈ 3000–80,000 g/mol for common species) is the key factor, with lower molecular weights degrading more rapidly [8,56]. Two effects are generally proposed to drive this: the higher concentration of carboxyl chain ends, which autocatalyse ester hydrolysis, and the smaller number of chain scissions needed before fragments fall below the threshold for dissolution and cellular uptake (M_n_~5000 g/mol) [18]. High molecular weight PCL degrades more slowly because it reaches that threshold later, and because water access to the backbone esters is limited by the polymer’s hydrophobicity and high crystallinity rather than by chain mobility.

To systematically dissect these effects, recent investigations have focused on isolating the role of initial chain length and molecular weight distribution. Lykins et al. [23] established a matrix of PCL films by blending distinct fractions to yield a continuous molecular weight spectrum for evaluation under accelerated acidic hydrolysis (4 M formic acid). Their results showed that although the degradation rate and water permeability remained largely constant for all blends, the average molecular weight determined the threshold for structural fragmentation; in particular, the inclusion of up to 50 wt% of a low M_n_ fraction halved the degradation time without causing any deterioration in the initial mechanical properties. In contrast, Hou et al. [57] evaluated 3D-printed scaffolds across a specific M_n_ spectrum exposed to highly alkaline accelerated conditions (5 M NaOH at 37 °C). Under this aggressive surface-mediated erosion regime, mass loss followed a uniform linear trajectory independent of the initial M_n_. However, lower molecular weight scaffolds showed increased hydrophilicity, enhanced cell proliferation and differentiation, and better mechanical properties (before degradation) resulting from higher polymer crystallinity. Similarly, Ma et al. [58] investigated the enzymatic erosion of PCL films utilizing *Candida antarctica* lipase in PBS (Phosphate-Buffered Saline) at 45 °C. Although the overall mass-loss kinetics were largely independent of the initial molecular weight, higher-molecular-weight films developed larger and progressively deeper surface pores. Ma et al. also reported a decrease in crystallinity and thermal stability, indicating substantial disruption of both amorphous and crystalline domains, while the principal chemical structure of PCL remained unchanged. This crystallinity trend contrasts with the preferential removal of amorphous regions and the resulting increase in crystallinity reported by Jenkins and Harrison [59], highlighting the sensitivity of morphological evolution to the enzyme type and degradation conditions.

Beyond absolute molecular weight metrics, the polydispersity index (PDI = M_w_/M_n_) dictates the homogeneity of the degradation profile. An increased PDI indicates a broad macromolecular mass distribution, introducing a significant population of short-chain oligomers that possess a high density of reactive carboxyl end groups (-COOH). These shorter chains act as highly mobile elements within the matrix. Rapid early hydrolysis of these low-molecular-weight fragments releases acidic byproducts locally, lowering the microenvironmental pH and inducing an autocatalytic acceleration of cleavage along adjacent long chains [19]. This heterogeneous mass loss complicates the predictability of implant longevity in vivo. For instance, Dias et al. [60] monitored the evolution of PDI in electrospun PCL scaffolds under decoupled hydrolytic (PBS) and enzymatic regimes. In the initial phase of exposure, hydrolysis in molecular weight resulted in a slight reduction in molecular weight accompanied by a broadening of the PDI, which is a characteristic symptom of random chain cleavage occurring throughout the matrix. Conversely, enzymatic degradation was localized strictly to the biomaterial surface, leaving both the bulk molecular weight and PDI metrics essentially static, thereby demonstrating that short–term PDI evolution is highly dependent on the underlying erosion mechanism.

Crystalline morphology constitutes an additional structural factor governing the degradation rate of PCL and is closely linked to molecular weight and thermal history. In particular, lower-molecular-weight chains may exhibit a greater propensity for crystallization, although the resulting degree of crystallinity also depends strongly on processing conditions and thermal history [61]. Jenkins and Harrison [59] found a loss of enzymatic mass, indicating that the apparent effect of chain length may be partially confounded by differences in crystal morphology. Therefore, molecular weight should not be considered independently of the initial crystalline structure of the material. However, the subsequent evolution of crystalline degradation is condition-dependent: preferential removal of amorphous regions may increase the relative crystalline fraction, whereas under more aggressive enzymatic conditions substantial disruption of crystalline domains may instead decrease crystallinity.

A comparative overview of the principal polymer-related parameters governing PCL degradation is provided in Table 2.

### 2.1. Material Composition

#### 2.1.1. The Effect of Additives on PCL Degradation

Composites based on polycaprolactone are widely investigated due to the polymer’s high biocompatibility, biodegradability, and regulatory acceptance, as well as its favorable processing characteristics. As a semicrystalline aliphatic polyester with a relatively low melting temperature, PCL can be readily processed using conventional thermal techniques without significant macromolecular degradation, making it particularly attractive for advanced biomaterial engineering. However, neat PCL exhibits slow intrinsic degradation kinetics, with complete in vivo resorption typically extending over two to four years depending on the implantation site and morphological structure. To overcome the limitations associated with its low mechanical stiffness and pronounced hydrophobicity, PCL is frequently modified via blending with secondary polymers or the incorporation of functional additives and plasticizers. These compositional modifications allow for the precise tailoring of macromolecular flexibility, crystallinity, and water permeability, which directly dictate the downstream erosion profile. Specifically, the integration of secondary polymeric phases or low-molecular-weight modifiers modulates water diffusion, chain mobility, and phase morphology. Table 3 provides a comprehensive overview of the polymeric additives incorporated into PCL matrices, detailing their specific functions, effects on key physicochemical properties, degradation behavior, and typical biomedical applications.

Among hydrophilic modifiers, poly(ethylene glycol) (PEG) is widely utilized to convert hydrophobic PCL into a functional, amphiphilic copolymeric or physically blended system is often proposed to exhibit enhanced cell affinity and accelerated degradation profiles depending on the specific formulation [62]. The incorporation of PEG significantly alters the degradation kinetics by augmenting the hydrophilic character of the bulk matrix. For instance, Hoque et al. [63] demonstrated that under accelerated hydrolytic conditions (5 M NaOH at 37 °C), 3D-printed PCL–PEG scaffolds exhibited accelerated mass loss and rapid internal porosity evolution compared to neat PCL controls, a phenomenon driven by enhanced water penetration into the PEG-rich domains. Similarly, Wang et al. [64] reported that in phosphate-buffered saline supplemented with *Aspergillus oryzae* lipase, PCL–PEG copolymers underwent accelerated enzymatic cleavage, manifesting steeper weight loss trajectories and rapid molecular weight reduction. This kinetic acceleration is fundamentally mediated by increased macromolecular chain mobility and the physical leaching of the soluble PEG phase, which establishes an interconnected porous network that increases the surface area accessible to hydrolytic and enzymatic attack.

The compounding of PCL with other biodegradable polyesters, such as poly(lactic acid) (PLA), introduces distinct degradation dynamics governed by phase morphology. Engler et al. [65] showed that erosion was highly sensitive to phase continuity, crystallinity, and the spatial distribution of the two polymers. Preferential degradation of the more accessible or hydrolytically susceptible domains generated microvoids and interfacial defects, thereby facilitating water transport through the phase-separated matrix. Although these findings cannot be directly extrapolated to physiological conditions, the underlying morphology-mediated mechanism may remain relevant to biomedical PLA/PCL systems, in which degradation of one phase can increase porosity and expose previously shielded regions of the other. The extent of this effect in vivo will nevertheless depend on temperature, fluid composition, enzymatic and cellular activity, implant geometry, and the local tissue environment [66].

To concurrently enhance cell affinity and introduce antibacterial functionality, PCL is frequently formulated as physical blends or composite networks with naturally derived polysaccharides such as chitosan [67]. This strategy couples the mechanical durability of the synthetic polyester with the hydrophilicity and biological functionality of the natural polymer. Gámiz-González et al. [68] showed that chitosan–PCL networks absorbed substantial amounts of water and underwent markedly faster degradation under lipase-mediated conditions than under purely hydrolytic conditions. The enhanced susceptibility of these systems was associated primarily with their increased water uptake, swelling, and improved accessibility of the degradable PCL domains. Consistent with this trend, Wan et al. [69] reported that porous PCL/chitosan scaffolds exposed to PBS and enzymatic media for up to 10 weeks exhibited progressively faster degradation with increasing chitosan content. Thus, chitosan is proposed to accelerate the apparent degradation of PCL-based systems mainly by increasing hydration, porosity, and transport through the polymer network, while the precise contribution of its amino groups to local acid–base regulation remains highly dependent on composition, protonation state, interfacial compatibility and degradation conditions.

Recently, carbon-based nanomaterials, particularly graphene oxide (GO), have attracted attention as potential modifiers of both the mechanical and degradation behavior of PCL-based composites. Although oxygen-containing groups on GO can interact with the ester groups of PCL’s, the intrinsic hydrophilic–hydrophobic mismatch between GO and PCL often limits homogeneous dispersion and may promote agglomeration. Therefore, mechanical reinforcement is typically observed only when GO is incorporated at appropriate concentration and its interfacial compatibility is improved, for example, through surface functionalization [70]. Campillo-Fernández et al. [71] and Martínez-Ramón et al. [72] systematically evaluated the stability of PCL/GO composites (0.1, 0.2, and 0.5 wt%) across divergent chemical and biological environments, including extreme pH regimes (pH 1 and 13) and lipase-supplemented physiological buffers (pH 7.4). Under highly alkaline conditions, the surface chemistry of the GO sheets was proposed to catalyze the hydrolytic cleavage of PCL, triggering complete structural dissolution of the 0.5 wt% GO composite within 72 h. In stark contrast, under enzymatic testing with *Pseudomonas* lipase, the embedded GO sheets functioned as physical stabilizers, sterically hindering enzymatic binding and retarding the degradation rate in a filler-concentration-dependent manner. These dual profiles underline the role of GO as a responsive degradation modulator whose final kinetic impact is governed by the chemical or biological nature of the surrounding medium.

**Table 3:** Overview of polymeric additives incorporated into poly(ε-caprolactone) matrices, including their functions, effects on key properties, degradation behavior, and typical biomedical applications.

Additive Name	Function in PCL Matrix	Key Properties Affected	Degradation Behavior	Application	References
Polylactic acid (PLA)	Increases stiffness and structural stability of the composite.	Increases stiffness and reduces ductility; effects depend on morphology and composition.	Phase-dependent degradation may generate pores and improve water transport through the blend.	Tissue engineering scaffolds, biodegradable implants, 3D printing.	[65,66]
Polyethylene glycol (PEG)	Hydrophilic modifier and pore-forming component.	Increases water uptake and chain mobility.	Accelerates degradation due to enhanced water diffusion.	Drug delivery systems, hydrogels, tissue engineering.	[63,64]
Graphene oxide (GO)	Reinforcement additive enhancing structural integrity and multifunctional performance	May improve stiffness and strength if well dispersed; agglomeration may limit reinforcement.	Environment-dependent: accelerates alkaline hydrolysis but retards lipase-mediated degradation.	Tissue engineering scaffolds, conductive biomaterials, drug delivery systems, sensors, and advanced composite materials.	[70,71,72]
Polyethylene oxide (PEO)	Improves processability and fiber formation.	Enhances flexibility and hydrophilicity reduces crystallinity.	Promotes faster degradation due to its water-soluble nature.	Electrospun scaffolds, drug delivery, biomedical coatings.	[73,74,75]
Poly-L-lactic acid (PLLA)	Provides reinforcement and improves structural rigidity.	Increases tensile strength and modulus; decreases ductility.	Slower than PLA but faster than PCL; allows turning of degradation profile.	Bone tissue engineering, load-bearing scaffolds, implants.	[76]
Chitosan	Enhances bioactivity and introduces antibacterial properties.	Improves hydrophilicity, cell adhesion, and antimicrobial activity; may reduce mechanical strength.	Accelerates apparent degradation by increasing hydration and porosity.	Wound healing, tissue engineering, drug delivery.	[68,69]
Poly(lactic-co-glycolic acid) PLGA	Modifies degradation rate and enhances bioresorbability.	Improves biodegradability and tunability of properties; moderate mechanical strength.	Faster and controllable degradation depending on lactic/glycolic ratio	Drug delivery systems, resorbable scaffolds, biomedical implants.	[76,77]

#### 2.1.2. The Effect of Bioceramic Additives on PCL Degradation

The fabrication of organic–inorganic biocomposites via the incorporation of bioactive ceramics, such as hydroxyapatite (HA), β-tricalcium phosphate (β-TCP), and bioactive glasses (BG), is a common strategy to overcome the intrinsic limitations of neat PCL, namely its hydrophobic character, physiological inertness, and modest mechanical stiffness [78]. The integration of these rigid inorganic phases fundamentally reinforces the viscoelastic polymer matrix; for instance, compounding PCL with 20–30 wt% β-TCP substantially augments the compressive modulus (E_c_) and load-bearing resistance, yielding a construct that structurally mimics the biphasic architecture of native osseous tissue [79,80,81]. For functional bone tissue engineering, an ideal scaffold must undergo structural resorption at a kinetic rate that is synchronized with de novo osteogenesis in vivo [82]. This temporal coordination ensures that the artificial matrix provides initial mechanical competence before progressively transferring physiological loads to the regenerating bone architecture [78]. A detailed, comprehensive overview of various bioceramic additives utilized in PCL-based composites, highlighting their primary functions, distinct effects on key physical and mechanical properties, specific degradation behavior, and typical biomedical applications, is systematically summarized in Table 4.

The addition of bioceramic fillers modulates the hydrolytic degradation profile of the surrounding PCL matrix, primarily by altering water diffusion kinetics and localized microenvironmental pH. Chouzouri and Xanthos [83] demonstrated that the incorporation of 30 wt% calcium silicate (CS) or 45S5 bioactive glass significantly elevates the water absorption capacity of PCL composites, thereby accelerating the random hydrolytic cleavage of the ester bonds along the polyester backbone. Crucially, the alkaline dissolution products of these ceramics are thought to act as internal buffering agents, neutralizing the acidic carboxyl end groups (-COOH) liberated during autocatalytic PCL breakdown, which stabilizes the local microenvironment. Liu et al. [84] confirmed this mechanism by incorporating a reactive CS phase into high-molecular-weight PCL scaffolds, which established an alkaline microenvironment in both Tris-HCl and Dulbecco’s Modified Eagle Medium (DMEM). Although the dissolution of the CS phase accelerates ester hydrolysis via localized pH elevation, the protective PCL coating dampens this initial reactivity, ensuring the retention of structural and mechanical integrity over a critical four-week window. Similarly, Larrañaga et al. [85] and Gritsch et al. [86] investigated the degradation pathways of PCL composites filled with 15–30 vol% bioactive glass (45S5 or Si/Ca formulations) in PBS and SBF (Simulated Body Fluid) at 37 °C. Their findings indicated that the basic ions released from the 45S5 BG phase suppressed the accelerated bulk autocatalysis typically driven by accumulated acidic oligomers. Furthermore, the presence of the bioactive glass induced the rapid nucleation and growth of a biomimetic bone-like apatite layer on the scaffold surface within the initial days of incubation, enhancing interfacial bioactivity.

Hydroxyapatite, particularly when engineered as a nanomaterial (nHA), is widely utilized to tailor the resorption kinetics of PCL matrices. Jiao et al. [87] employed melt differential 3D printing to construct interbody fusion cages from PCL and HA mixtures (25–40 wt%) using two distinct polymer molecular weights. Assessments in SBF at 37 °C revealed that increasing the HA mass fraction accelerated the absolute degradation rate. Interestingly, cages formulated with the higher molecular weight PCL exhibited accelerated mass loss compared to their counterparts at equivalent HA loadings, with a 30 wt% HA formulation identified as the optimal threshold to balance printability, mechanical strength, and degradation longevity specifically for their melt-extruded experimental system. This accelerative trend was corroborated by Diaz et al. [88], who established that loading PCL with nHA (10, 30, and 50 wt%) was associated with substantial weight loss and a pronounced reduction in molecular weight after 16 weeks in SBF, a phenomenon directly linked to enhanced initial water uptake at the organic–inorganic interface. To rapidly screen these formulations, Doyle et al. [89] utilized an accelerated hydrolytic environment (5 M NaOH at 37 °C) to compare PCL scaffolds of varying molecular weights doped with nHA (10, 30, and 50 *w*/*v*%). The findings consistently demonstrated that nHA inclusion catalyzes ester cleavage relative to the neat polymer, confirming that the inorganic volume fraction serves as a tunable dial for degradation control under these accelerated conditions [86].

As a synthetic, osteoconductive ceramic with a definitive calcium-to-phosphorus (Ca:P) ratio of 1.5, β-tricalcium phosphate represents another key degradable modifier for bone scaffolds. Comprehensive investigations by Lam et al. [90,91] demonstrated that incorporating 20 wt% β-TCP into PCL scaffolds accelerates hydrolytic degradation under both physiological (PBS, pH 7.4) and accelerated alkaline (5 M NaOH) conditions at 37 °C. While the neat PCL controls required up to six weeks for complete mass dissolution in the alkaline medium, the PCL–TCP composites underwent complete breakdown within 54 h. These degradation times are specific to the aggressive alkaline testing environment (5 M NaOH) used in this study and should not be extrapolated directly to in vivo timelines. The precise degradation mechanism was found to be highly dependent on the surrounding environment: a surface-mediated erosion regime dominated under aggressive alkaline exposure, whereas a slow, homogeneous bulk hydrolysis occurred in physiological PBS. These insights confirm that the inclusion of a degradable β-TCP phase allows for the precise tuning of scaffold resorption kinetics and surface bioactivity without compromising the functional stability or the long-term recrystallization capacity of the remaining PCL framework.

**Table 4:** Overview of bioceramic additives used in PCL-based composites, highlighting their functions, effects on key properties, degradation behavior, and typical biomedical applications.

Additive Name	Function in PCL Matrix	Key Properties Affected	Degradation Behavior	Application	References
Hydroxyapatite (HA)	Enhances the bioactivity of the bone mineral phase.	Improves stiffness, osteoconductivity, and surface bioactivity.	May either accelerate or slightly slow down degradation depending on HA content and dispersion; increased hydrophilicity and porosity often promote faster degradation, while barrier effects can limit water penetration	Bone tissue engineering, coatings, orthopedic implants.	[87,92,93,94]
Nano-hydroxyapatite (nHA)	Provides high surface area for enhanced biological interactions.	Enhances mechanical properties, cell adhesion, and proliferation.	Can accelerate degradation compared to HA due to higher surface area, promoting water uptake and interfacial hydrolysis.	Bone regeneration, nanocomposite scaffolds, drug delivery.	[88,89,95]
Biphasic Calcium Phosphate (BCP)	Combines stability of HA and resorbability of TCP.	Balanced mechanical strength and bioactivity.	Provides tunable degradation depending on HA/β-TCP; higher TCP content increases degradation rate.	Bone grafts, tissue engineering scaffolds.	[96,97,98]
α-Tricalcium Phosphate (α-TCP)	Highly reactive phase promoting rapid bone formation.	Moderate mechanical properties; high bioactivity.	Accelerates degradation due to its high solubility and rapid resorption.	Bone cements, fast-resorbing scaffolds.	[99]
β-Tricalcium Phosphate (β-TCP)	Enhances bioresorbability and supports bone regeneration.	Provides moderate mechanical reinforcement and high osteoconductivity.	Moderately accelerates degradation, enabling more controlled and predictable resorption compared to other TCP phases.	Bone tissue engineering, resorbable scaffolds, bone graft substitutes.	[80,90,91]
Calcium Silicate (CS)	Promotes bioactivity and stimulates mineralization.	Improves mechanical strength and enhances osteogenic activity.	Enhances degradation due to ionic dissolution increased local pH, which promotes hydrolytic breakdown of PCL.	Bone tissue engineering, bioactive scaffolds.	[83,84]
Bioactive Glass (BG)	Stimulates bonding with bone and soft tissue.	Enhances bioactivity, ion release, and osteostimulation.	Accelerates degradation through ion release and increased hydrophilicity, promoting polymer hydrolysis.	Bone regeneration, coatings, tissue engineering.	[85,86]

### 2.2. Geometry and Morphology

The degradation kinetics of polycaprolactone are governed by a multifactorial process in which the geometric design—particularly architecture, porosity, and surface-to-volume ratio (SA/V)—exerts a decisive influence on the rate of hydrolytic cleavage, often outweighing the internal chemical composition alone [33]. Under physiological conditions, the fundamental degradation mode of PCL is characterized by bulk erosion, which involves the random scission of ester bonds uniformly throughout the entire cross-section of the polymer matrix [19]. However, the spatial dimensions of the construct heavily modulate how this mechanism practically unfolds. In massive, non-porous monolithic forms, physical geometry severely restricts the outward diffusion of degradation products. This limitation induces a profound autocatalytic effect, wherein acidic carboxylic groups accumulate within the core of the material, dropping the local pH and significantly accelerating internal chain scission compared to the outer boundaries [33,100]. Consequently, this diffusion barrier enforces a heterogeneous “skin-core” degradation model in massive, non-porous constructs; the number-average molecular weight decreases rapidly at the center of the sample, while the surface remains structurally stable due to continuous neutralization by the surrounding buffer medium [20,100].

Downscaling the polymer geometry into thin films significantly alters these mass transport dynamics, enabling a more uniform degradation profile. Because the diffusion pathway for oligomers is exceptionally short in thin films, internal autocatalysis is minimized, and degradation proceeds homogeneously throughout the volume [54]. At this scale, the erosion pattern is highly sensitive to the surface micro-geometry and the specific degree of crystallinity induced during processing. For instance, the surface spherulitic morphology determines the spatial progression of mass loss, as degradative enzymes preferentially target the less ordered, vulnerable amorphous regions located between the dense crystalline spherulites [28].

When transitioning to three-dimensional porous scaffolds—particularly those developed via additive manufacturing—the structural architecture introduces a notable geometric effect. High porosity and extensive pore interconnectivity facilitate fluid percolation and the rapid flushing out of acidic degradation byproducts [29,33]. Paradoxically, this open network prevents the onset of severe autocatalysis, allowing porous scaffolds to exhibit a slower rate of molecular weight drop over time compared to solid monolithic blocks [32,91]. To model these kinetics, the “dimensionless surface area” has been established as a critical geometric parameter, enabling a linear prediction of filament mass loss regardless of the specific layer lay-down pattern [101]. Despite this, degradation within these architectures remains topologically non-uniform; due to the local reduction in the SA/V ratio, hydrolytic degradation proceeds slower at fiber intersections (junctions) than along regular, fully exposed segments of the printed fibers [101,102].

At the micro- and nanoscale, spatial architecture and structural geometry serve as critical regulators of PCL degradation kinetics. In particulate systems, this process follows a distinct two-phase mechanism. During the initial phase, which lasts approximately three years, PCL microparticles undergo strictly isovolemic degradation. Throughout this period, hydrolytic chain scission causes a progressive drop in molecular weight, yet the core particle volume and geometric diameter remain virtually unchanged [103]. The onset of the second phase, occurring around the fourth year, is characterized by bulk degradation driven by internal autocatalysis. This structural transition manifests as pronounced surface cracking, particle fragmentation, and a significant reduction in overall size [27,103]. To modulate these kinetics, modern processing designs introduce controlled internal porosity into 25–50 µm microspheres. This porous morphology drastically enhances the accessible specific surface area, promoting a gradual, highly uniform hydrolytic profile while maintaining long-term biocompatibility [27].

Analogously, the degradation behavior of fibrous matrices can be precisely programmed by tailoring fiber geometry and morphology during fabrication. The spatial properties and fine-tuning of electrospun nanofibrous scaffolds are extremely sensitive to manufacturing history, such as the exact dissolution time of the polymer in acidic hydrolytic solvents prior to spinning [31]. Furthermore, degradation rates can be strategically controlled by engineered polyblends that combine high- and low-molecular-weight PCL fractions within individual nanofibers [104]. In these multi-phase systems, the low-molecular-weight domains degrade preferentially, dynamically altering the scaffold’s local porosity and fiber morphology over time, while the high–molecular–weight matrix preserves the structural integrity of the construct [104].

To provide a comprehensive overview, the critical degradation descriptors across these different structural dimensions and geometries are systematically contrasted in Table 5.

In summary, the geometry and morphology of PCL constructs directly dictate their degradation mode and kinetics. This is clearly demonstrated by the porosity paradox: despite a larger surface area, highly interconnected 3D scaffolds lose molecular weight slower than monolithic blocks, as their open architecture prevents oligomer entrapment and subsequent internal autocatalysis [32,85].

Furthermore, downscaling biomaterial dimensions (from bulk blocks → 3D printed strands → fibers → nanoparticles) triggers a decisive mechanism shift driven by the SA/V scale. This shift reflects a transition from unpredictable bulk erosion to predictable, diffusion-controlled surface erosion [101,105].

However, this process is non-uniform within complex architectures due to structural junction effects, where printed filament intersections degrade slower due to a locally lower SA/V ratio [101].

Finally, these kinetic profiles are strongly modulated by processing-induced surface constraints; manufacturing methods (e.g., electrospinning vs. molding) alter surface topography and hydrophobicity, meaning that even high-SA/V nanofibrous meshes can temporarily delay water entry and initial hydrolytic cleavage [106].

### 2.3. Environmental Conditions

The degradation of biodegradable polymers is governed not only by their intrinsic physicochemical properties but also by the conditions of the surrounding medium. Its composition, pH, and temperature modulate water uptake, hydrolysis, and the removal of degradation products, ultimately shaping the rate and mechanism of PCL degradation [107].

Studies of hydrolytic degradation in biomedical engineering most often focus on PBS or SBF solutions. PBS serves as a straightforward buffer that helps sustain pH and osmotic balance for biological cells, approximating the ionic composition of extracellular fluid, though it lacks its proteins, cells, and dynamic flow. In contrast, SBF is a more intricate solution containing ion concentrations (such as Ca^2+^ and Mg^2+^) formulated to approximate the inorganic composition of human blood plasma. It is frequently utilized for evaluating the in vitro bioactivity of biomaterials [108], though it lacks the proteins, cells, and dynamic flow of true physiological environments. Accordingly, the choice of degradation medium should be aligned with the intended biological environment and the specific degradation mechanism under investigation. The principal selection criteria are summarized in Table 6.

Idaszek et al. [111] compared degradation in PBS and SBF to distinguish between purely hydrolytic degradation (PBS) and more physiologically relevant conditions where ion exchange and mineral deposition can occur (SBF), better mimicking the in vivo environment. They observed that while mass loss trends were initially similar in both media, samples in SBF showed additional effects such as calcium phosphate precipitation and even mass gain at later stages, highlighting the influence of bioactive processes on degradation behavior.

However, while these standardized abiotic environments provide critical baseline profiles of pure hydrolytic or mineralizing kinetics, they fail to replicate the multi-component complexity of localized physiological microenvironments. In vivo, biomaterials are rarely exposed to static, chemically isolated buffers; instead, they interact with dynamic biofluids characterized by fluctuating ion gradients, metabolic waste products, organic macromolecules, and microbial colonizers. To bridge this translational gap, recent investigative paradigms have shifted toward evaluating poly(caprolactone) degradation within site-specific simulated or ex vivo body fluids. These specialized environments alter the interfacial degradation mechanisms, transforming a simple chemical hydrolysis into a complex process governed by bio-macromolecular fouling, enzymatic erosion, or surface encrustation.

According to studies by Łysik et al. [100,112], PCL degradation in simulated artificial saliva primarily follows a bulk degradation mechanism, meaning the hydrolysis occurs throughout the entire volume of the material. Initially, the adsorption of mucins from the saliva forms a protective coating on the polymer surface that slightly slows the degradation process compared to standard saline solutions. However, the formation of a bacterial biofilm, particularly *Streptococcus mutans*, significantly accelerates this process by promoting more rapid polymer breakdown. These combined factors eventually lead to a measurable reduction in molecular weight and the deterioration of the polymer’s mechanical strength.

A similarly intricate biological environment dictates the performance of PCL in urological applications, such as biodegradable stents or chemical delivery systems. Deka et al. [22] evaluated the structural durability of PCL films exposed to extreme urological shifts, specifically focusing on unconcentrated and concentrated alkaline urine stabilized with calcium hydroxide (Ca(OH)_2_). Under these highly alkaline conditions, intended to recover nutrients or control solute release, PCL exhibited distinct morphological vulnerabilities. The prolonged interaction with elevated ionic strength and specific chemical additives accelerated the degradation cascade, causing substantial alterations in the physical framework and surface topography of the thin films, demonstrating that chemical stabilization parameters within urinary environments can dramatically alter the structural lifecycle of the polyester.

The transition of PCL applications into oral or gastrointestinal delivery systems further intensifies the catalytic breakdown of the polymer due to specialized physiological fluids. Chang et al. [113] conducted an ex vivo evaluation of PCL films within various localized digestive fluids. Their morphological assessments revealed that while PCL maintains an exceptionally smooth surface upon fabrication, its incubation in different digestive secretions triggers highly divergent degradation pathways. While simple hydrolytic cleavage persisted as a baseline mechanism, the multi-component nature of the digestive juices–varying in pH and biomacromolecular content–induced pronounced localized variations in erosion rates across different segments of the gastrointestinal system.

To systematically isolate these gastrointestinal mechanisms, researchers utilize defined simulation models. Tarvainen et al. [114] investigated the degradation and mass loss kinetics of PCL and modified 2,2′-bis(2-oxazoline)-linked PCL films and bars within simulated gastric fluid (SGF, pH 1.2, containing pepsin) and simulated intestinal fluid (SIF, pH 7.5, containing pancreatin). Their size exclusion chromatography and weight loss data established that while the strongly acidic, pepsin-rich SGF primarily drives chemical hydrolysis, the introduction of pancreatin in SIF dramatically alters the erosion mode. The enzyme complexes in pancreatin, which are thought to be sterically restricted from infiltrating the densely packed crystalline domains of the polymer, were shown to accelerate mass loss via a predominantly surface-confined erosion mechanism. This underscores how specific biochemical catalysts can override standard hydrolytic timelines.

This enzymatic dominance within the intestinal tract has been corroborated by both in vivo and mechanical testing models. Peerlinck et al. [115] investigated the precise impact of small intestinal fluid exposure on the mechanical performance of PCL, demonstrating that the enzymatic cocktail induces rapid surface alterations that directly impair the tensile and viscoelastic stability of the polymer. To capture the full physiological feedback loop, Chang et al. [50] advanced this to an in vivo gastrointestinal model, tracking PCL films passing through the actual stomach and intestinal tracts of living subjects. Their infrared spectroscopic analysis mapped a progressive rise in the crystallinity index over a 28-day period, confirming that the amorphous regions of PCL are preferentially consumed by the combined action of acid-catalyzed bulk hydrolysis and enzyme-driven surface erosion in this gastrointestinal model. Furthermore, histological evaluations revealed that the degradation products elicited minor, transient mucosal and goblet cell swelling, validating that while the specific gastrointestinal environment aggressively accelerates PCL mass loss, the interfacial tissue response remains biocompatible.

The accelerating effects observed across these diverse physiological fluids–ranging from microbial breakdown in saliva to pancreatin action in the intestines–reflect a consistent pattern: the shift from slow abiotic hydrolysis to rapid material degradation is largely driven by biocatalytic intervention. Whether mediated by extracellular enzymes secreted by a bacterial biofilm or by the body’s native digestive proteins, biocatalysts introduce high substrate specificity and drastically lower the activation energy required for ester bond cleavage. Because these large enzymatic macromolecules are sterically hindered from infiltrating the dense, crystalline regions of PCL, they preferentially target the loose, amorphous chains at the material’s exterior. This restricts the degradation front, often forcing a condition-dependent transition from uniform bulk hydrolysis to surface-confined erosion. To systematically evaluate how these distinct biological agents govern the polymer lifecycle, the active environments can be categorized based on their underlying catalytic systems.

#### 2.3.1. Mechanistic Insights into Biocatalytic Systems

The diverse experimental configurations compiled in Table 7 reveal that biocatalytic degradation of poly(caprolactone) is not merely a function of enzyme concentration, but a phenomenon governed by the origin of the biocatalyst, the structural architecture of the polymeric matrix, and the surrounding medium.

#### 2.3.2. Lipase-Mediated Hydrolysis and Structural Constraints

Lipases represent the most extensively quantified class of biocatalysts for PCL degradation, operating primarily through highly localized surface-confined ester bond cleavage. This surface-dominated erosion is clearly demonstrated by *Pseudomonas* lipases, which can completely degrade highly crystalline 3D-printed scaffolds or electrospun nanofibers within hours to days while leaving the molecular weight of the remaining inner core largely intact [116,117,118]. However, the efficiency of this interfacial attack is strictly limited by the physical state of the polymer chains. As highlighted by studies utilizing *Rhizopus* lipases and *Burkholderia cepacia*, the rate of enzymatic cleavage is inversely proportional to the matrix draw ratio and global crystallinity [60,119]. Because the bulky catalytic pockets of these macromolecular enzymes are sterically hindered from entering the dense, tightly packed crystalline lamellae of PCL, they preferentially target the highly accessible, disordered amorphous regions. Interestingly, as shown in *Burkholderia cepacia* systems, this preferential amorphous erosion temporarily drives up the relative crystallinity index of the remaining matrix during the initial weeks of incubation before progressive disruption of the crystalline domains finally takes place [60].

Deviations from this standard surface erosion pathway occur when the spatial distribution of the enzyme is altered or when non-aqueous environments are introduced. Embedding lipases directly into the polymer matrix–such as *Lactobacillus plantarum* or CALB–shifts the degradation profile toward intra-matrix hydrolysis. In these self-degrading or embedded configurations, the biocatalyst induces micro- to micron-scale surface and bulk porosity (2–3 µm), actively modifying the 3D architecture from within [35,37]. Furthermore, under low-water conditions (e.g., organic solvents like toluene), the catalytic behavior of lipases like CALB shifts toward an intramolecular “back-biting” mechanism, converting linear polymer chains into cyclic [37,38] oligomers (di-, tri-, and tetracaprolactone) and expanding the synthetic and recycling utility of the enzyme.

#### 2.3.3. Bacterial and Fungal Depolymerization Kinetics

When moving from isolated enzymes to living microbial systems, the degradation kinetics become coupled with cellular metabolism, where PCL serves as the primary or sole carbon source. Bacterial isolates from extreme environments, such as the thermophilic *Ralstonia* sp. strain MRL-TL (optimal at 50 °C) and the robust *Brevundimonas* sp. strain MRL-AN1, secrete specialized extracellular depolymerases (e.g., serine hydrolases) that readily breach the hydrophobic surface of solvent-cast films, resulting in deep topographical pitting and mass losses exceeding 80% within a mere 10 days [70,120,121]. To overcome the natural resistance of high-molecular-weight PCL, methodological interventions such as UV-pretreatment have been successfully employed, as seen in *Alcaligenes faecalis* models, where photo-oxidation breaks down long polymer chains into shorter, hydrophilic segments, enabling complete material consumption over approximately 60 days [75,122].

Fungal degradation pathways, predominantly characterized by *Aspergillus* and *Fusarium* species, introduce distinct macromolecular and physiological environmental dynamics. *Aspergillus fumigatus* systems confirm a surface-dominated erosion mechanism where the addition of inorganic fillers (SiO_2_) acts as a structural catalyst, increasing the effective surface area available for fungal hyphae attachment and subsequent enzymatic secretion [123]. Moreover, fungal metabolism actively alters the local microenvironment; metabolic processing by *Aspergillus niger* induces sharp localized pH drops, which in turn chemically catalyze ester cleavage, driving the fastest degradation rates when the polymer is positioned in restricted, high-moisture zones within agar matrices [124].

#### 2.3.4. Macromolecular Selectivity and Substrate Exploitation

The ultimate boundaries of biological specificity are exemplified by the interactions between PCL and non-specific proteases, such as Proteinase K (derived from *Tritirachium album*). In absolute contrast to lipases, Proteinase K exhibits high macromolecular bio-selectivity: it is essentially inert toward both the amorphous and crystalline domains of PCL, while preferentially targeting the amorphous phases of neighboring polyesters like poly(L-lactide) (PLLA) [125]. Rather than limiting its biomedical utility, this complete absence of direct degradation is strategically exploited in two ways: first, as a highly precise tool for morphological mapping in blended systems, and second, to selectively leach out PLLA from PLLA/PCL blends to manufacture highly porous, customized PCL scaffolds. Alternatively, indirect PCL degradation can be achieved by incorporating biodegradable biopolymer fillers; in PCL/gluten composites exposed to proteases, the enzyme digests the integrated protein phase, creating an interconnected porous network that substantially increases the surface area, thereby pre-activating the remaining PCL framework for accelerated abiotic or chemical hydrolysis [126].

**Table 7:** Classification of Biocatalytic PCL Degradation Systems.

Category	Biocatalytic System/Strain	Polymeric Matrix Configuration	Key Observations and Mechanisms	Reference(s)
Lipases	*Candida antarctica* Lipase B (CALB)	Crystal flakes, embedded matrices	Back-biting” mechanism in low-water environments; generation of cyclic oligomers (di-, tri-, tetracaprolactone). In embedded systems, it facilitates 3D intra-matrix hydrolysis, creating high-porosity structures	[44]
	*Pseudomonas lipases* (e.g., *P. cepacia*, *P. fluorescens*)	FDM 3D scaffolds, electrospun nanofibers, photopolymerized networks	Dominant surface erosion; maintains core molecular weight integrity while causing significant mass and mechanical property loss. Highly crystalline PCL can be fully degraded within 4–80 h depending on concentration	[21,49,59]
	*Lactobacillus plantarum* lipase	Self-degrading films (enzyme-embedded)	Creation of micron-scale surface and bulk pores (2–3 µm); mass loss correlates linearly with enzyme loading (2–8 wt%); highly effective under static, ambient conditions.	[37]
	*Rhizopus* lipases (e.g., *R. arrhizus*)	Drawn fibers, PCL-based copolymers	Degradation rate is inversely proportional to fiber draw ratio and crystallinity; the enzyme preferentially targets amorphous or less-ordered regions.	[116]
	*Mucor miehei*, *Candida rugosa*, and *Rhizopus delemar*	Dissolved PCL in Toluene	Among the three fungal lipases assessed in toluene, *Mucor miehei* was identified as the superior biocatalyst, achieving 70% PCL degradation within the first hour. In contrast, *C. rugosa* and *R. delemar* showed significantly lower activity under the same conditions. This high efficiency resulted in a drastic reduction in molecular weight and the successful generation of monomers and dimers.	[38]
Bacteria	*Ralstonia* sp. strain MRL-TL	Solvent-cast films (0.2 mm); Minimal Salt Medium	Thermophilic isolate from hot springs (optimum 50 °C); utilizes PCL as sole carbon source; produces a 50 kDa depolymerase (serine hydrolase). SEM shows deep pits and holes after 10 days.	[117]
	*Brevundimonas* sp. strain MRL-AN1	PCL pieces (80 kDa); (MSM)	secretes a 63.49 kDa depolymerase; stable at pH 5–9 and 20–45 °C. Achieves 80–85% mass loss of films within 10 days.	[35,118]
	*Alcaligenes faecalis*	High M_w_ films (80 kDa); Basal medium	Methodology involves UV-pretreatment of films to accelerate attack; bacteria preferentially degrade amorphous regions; 100% weight loss achieved in ~60 days.	[119,120]
	*Bacillus subtilis* (CCM 1999)	Thin films PCL and PCL/Graphite Oxide (GO) composites	Methodology uses nutrient broth supplemented with glucose; degradation is marked by surface cracking and a 10% mass loss over 42 days; secondary crystallization occurs in residual polymer.	[70,121]
	*Lysinibacillus* sp. 70038	Thin PCL Films	Methodology shows ~9 wt% degradation after 30 days; degradation proceeds via endo-type chain scission and surface erosion.	[75]
	*Burkholderia cepacia* lipase	Electrospun fiber mesh	Cleaves long aliphatic polyester chains via surface-confined ester bond hydrolysis, driving a 97.11% mass loss within 90 days. Preferential amorphous phase erosion temporarily shifts relative matrix crystallinity up to day 42, followed by progressive crystalline domain disruption.	[60]
Fungi	*Fusarium solani*	Powders PCL and films with varying M_w_ and crystallinity	High biodegradative activity. The degradation rate is inversely proportional to M_w_ and crystallinity; low molecular weight PCL exhibits the highest susceptibility to enzymatic cleavage.	[70,122]
	*Aspergillus niger*	Compression-molded PCL films	Methodological study on polymer position in agar; degradation is fastest when the film is in the “middle” position (13.89% mass loss) due to localized pH drops (acidic environment).	[127]
	*Aspergillus fumigatus*	Melt-pressed films with additives (SiO_2_, erucamide)	Rapid mass loss (up to 45 days) with minimal reduction in core molecular weight, indicating a surface-dominated erosion mechanism. SiO_2_ acts as a catalyst by increasing the effective surface area for fungal attack.	[128]
Proteinase K	Proteinase K (from *Tritirachium album*)	PLLA/PCL Blends	Absence of PCL degradation: Proteinase K exclusively targets amorphous domains of PLLA while leaving PCL (both crystalline and amorphous) entirely intact. This bio-selectivity is utilized for morphological mapping and creating porous PCL scaffolds.	[129]
	Proteinase K	PCL/Gluten Composites	Indirect degradation: Proteases digest the protein (gluten) filler, creating a porous PCL structure that is more susceptible to subsequent abiotic or chemical hydrolysis.	[130]

The degradation of polycaprolactone is often studied under soil burial conditions to simulate natural environmental exposure, allowing researchers to evaluate its biodegradability, interaction with native microbial communities, and long-term stability in realistic disposal scenarios, particularly given its applications in food-contact materials [124]. Akahori and Osawa [123] found that PCL–paper composites buried in soil for six months were extensively disintegrated, with some samples degrading in just two months. Similarly, Al Hosni et al. [125] observed that PCL discs buried for 10 months showed the highest degradation rates, suggesting their potential as a compostable plastic under these specific environmental criteria. Additionally, Darwis et al. [126] reported that radiation-crosslinked PCL buried for six months achieved 60% weight loss, indicating that the crosslinking structure does not significantly hinder its macroscopic disintegration; however, further studies would be required to verify complete metabolic mineralization under these conditions.

#### 2.3.5. The Effect of pH Medium on PCL Degradation

The human physiological environment has a pH ranging from 7.35 to 7.45 in arterial blood, which is considered slightly alkaline [131]. For this reason, standard incubation buffers, such as the previously mentioned PBS and SBF, have a pH range of 7–7.4 [110]. The literature includes studies on medical materials, including polycaprolactone, conducted across a range of pH conditions to simulate specific physiological and environmental settings, accelerate degradation processes, assess antimicrobial efficacy, and evaluate stability under pathological conditions such as infection, inflammation, or specialized tissue environments. While 7.4 represents blood pH, other body sites range from acidic (stomach pH 1.5–3.5) to slightly acidic (skin pH 4–6.5) [132].

According to research by Bartnikowski et al. [20], Campillo-Fernández et al. [71], and Lykins et al. [23], PCL in acidic conditions undergoes bulk degradation, which means the process happens throughout the entire material volume. This environment leads to a clear reduction in molecular weight and a loss of mechanical strength due to the breaking of ester bonds. Studies show that acidic hydrolysis is slower than basic degradation and typically features a long induction period before significant changes occur. The process is further driven by the release of acidic end groups that can accelerate the internal reaction over time.

More commonly, the literature describes degradation in an alkaline medium, typically induced by sodium hydroxide (NaOH) at varying molar concentrations ranging from 0.1 to 5 M [20,57,71,74,90,92,101,104,133,134]. According to Hou et al. [57] and Bartnikowski et al. [20], PCL in a basic environment undergoes surface erosion, meaning the degradation process is primarily limited to the outer layers of the material. Bartnikowski et al. [20] observed that this alkaline treatment increases surface hydrophilicity while the internal molecular weight and mechanical properties remain relatively unchanged. Hou et al. [57] showed that this leads to a linear decrease in mass and fiber diameter as the surface is gradually removed. Additionally, Niiyama et al. [104] suggests that blending different molecular weights of PCL allows for better control over this accelerated degradation speed.

#### 2.3.6. The Effect of Temperature on PCL Degradation

As mentioned earlier, PCL is a low-melting polymer. Therefore, selecting the polymer conditioning temperature is crucial. The most selected temperature for the degradation medium is 37 °C, which matches the temperature of the human body. Interestingly, researchers also use higher temperatures to accelerate the degradation rate. PCL exhibits a semi-crystalline architecture, characterized by crystalline domains that provide resistance against water ingress and deterioration. Elevated temperatures enhance the mobility of polymer chains and facilitate water infiltration into these crystalline regions, thereby accelerating the degradation of the ester linkages.

Hukins et al. [135] described the “10-degree rule” as a simple empirical method used in accelerated aging, stating that increasing the temperature by about 10 °C roughly doubles the rate of many chemical reactions in polymers. This enables researchers to simulate long-term material degradation over a much shorter time by conducting tests at elevated temperatures, while ensuring that the testing temperature remains sufficiently below the material’s melting point (56–64 °C) to avoid introducing unrealistic degradation mechanisms [136]. In practice, it is widely applied to rapidly predict how materials, such as those used in medical devices, will perform over extended periods of use. Erdal et al. [137] used an elevated temperature (e.g., 60 °C) to accelerate the inherently slow hydrolytic degradation of PCL. They clearly observed faster degradation at higher temperature, as significant morphological changes and degradation products appeared much earlier at 60 °C than at 37 °C. Pitt et al. [19] selected 40 °C for in vitro testing to better simulate physiological conditions. Their study showed that PCL degradation occurs at a comparable rate in vivo and in vitro at the same temperature.

Elevating the incubation temperature is essential for cultivation of thermophilic microorganisms, which have evolved to thrive in high-heat environments. These organisms require temperatures exceeding 37 °C to catalyze their metabolic processes and maintain cellular stability. An example is *Candida antarctica lipase,* which proves to be most effective at a temperature of 40–45 °C [58]. Studies have shown that *Streptomyces thermoviolaceus* [138] efficiently degrades polycaprolactone at around 45 °C, whereas *Brevibacillus thermoruber* [139] and thermophilic microbial communities exhibit effective degradation at temperatures above ~50 °C, highlighting their relevance due to the production of thermostable enzymes that enhance biodegradation efficiency and offer significant potential for industrial applications such as bioplastic waste management.

## 3. Consequences of Degradation—Changes in Material Properties

### 3.1. Molecular Weight Evolution

PCL degradation is reflected in the progressive evolution of its molecular-weight distribution. Changes in number-average molecular weight, weight-average molecular weight and polydispersity index provide direct insight into the kinetics of polymer chain cleavage and degradation. Gel permeation chromatography (GPC), also termed size-exclusion chromatography (SEC), is widely used to monitor these changes by separating polymer chains according to their hydrodynamic volume [140]. Coupling SEC with multi-angle light scattering (MALS) enables absolute molecular-weight determination without reliance on conventional calibration standards [141]. Less commonly, viscometry is used as a complementary approach to estimate the viscosity-average molecular weight (M_η_) through the Mark–Houwink–Sakurada relationship and to detect degradation-induced chain shortening [100].

**Figure 4:**
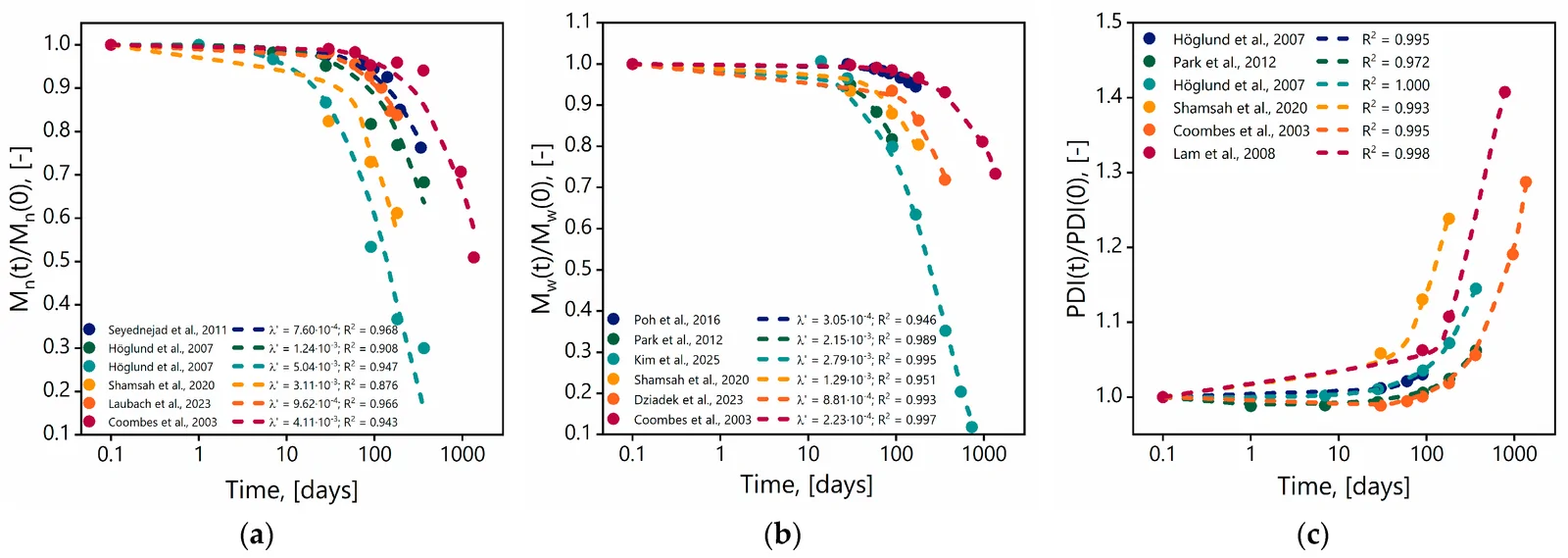
Kinetic evolution of PCL molecular weight parameters based on literature data. Data collected from studies conducted in SBF or PBS at 37 °C. (**a**) Normalized number-average molecular weight (M_n_), data from the literature [30,32,142,143,144]; (**b**) weight-average molecular weight (M_w_), data from the literature [27,142,143,145,146,147]; and (**c**) polydispersity index (PDI), data from the literature [32,134,142,143,147]. Dashed lines in panels (**a**,**b**) represent non-linear regressions using the first-order exponential decay model described by Equation (2). Dashed lines in panel (**c**) represent overall linear trend lines (Equation (3)) fitted to indicate the general direction of property changes over the degradation period.

The hydrolytic degradation kinetics of neat polycaprolactone under simulated physiological conditions (37 °C in PBS or SBF) are characterized by a multi-stage bulk erosion mechanism, as evidenced by the consolidated literature data. The temporal evolution of the number-average molecular weight, weight-average molecular weight, and the polydispersity index collectively delineates the progression from initial water diffusion to autocatalytic chain scission (Figure 4).

The compiled literature data reveal a broadly biphasic evolution of the molecular-weight characteristics of neat polycaprolactone under simulated physiological conditions. Despite considerable differences among the investigated materials, most normalized number-average molecular weight, M_n_(t)/M_n_(0), profiles remain close to unity during the initial period of incubation, followed by a progressively accelerated decline at longer exposure times. The onset and magnitude of this decrease vary substantially among the datasets, reflecting differences in initial molecular weight, processing history, crystallinity, specimen geometry, porosity, and surface–area–to–volume ratio. The most pronounced reduction was observed in one of the datasets reported by Höglund et al. [32], in which M_n_ decreased to approximately 20–30% of its initial value after prolonged incubation. By contrast, the materials investigated by Seyednejad et al. [30], Laubach et al. [144], and Shamsah et al. [143] retained approximately 60–90% of their initial Mn over the corresponding experimental periods. Coombes et al. [142] reported a more gradual but extended decline, reaching approximately 50% of the initial value at the longest incubation time. Overall, these differences indicate that PCL hydrolysis cannot be described by a single universal degradation timescale, even when the studies are conducted in PBS or SBF at 37 °C.

A similar time-dependent pattern is evident for the normalized weight-average molecular weight, M_w_(t)/M_w_(0). Most datasets exhibit only limited changes during the initial stages of incubation, followed by a more pronounced decrease after several tens or hundreds of days. However, the extent of the reduction differs considerably among studies. Kim et al. [27] reported the strongest long-term decline, for whom M_w_ decreased to approximately 10% of its initial value after more than 700 days. In the datasets of Park et al. [147], Shamsah et al. [143], Dziadek et al. [146], and Coombes et al. [142], the final normalized M_w_ values remained between approximately 0.6 and 0.8. These differences may arise from variations in specimen architecture and polymer microstructure, as well as from differences in the initial molecular-weight distribution. The delayed nonlinear decrease observed in many datasets is consistent with the progressive accumulation of hydrolytic chain scissions. Nevertheless, the normalized curves alone do not allow individual stages to be assigned unambiguously to water diffusion, oligomer release, or degradation of the crystalline regions.

The evolution of the normalized polydispersity index, PDI(t)/PDI(0), further demonstrates that hydrolytic degradation progressively alters the molecular-weight distribution of PCL. During the initial incubation period, most PDI values remain close to the initial level, with minor increases or decreases around unity. At longer degradation times, all datasets show a distinct increase in normalized PDI, although the onset and magnitude of this increase differ among studies. The highest value, approximately 1.4, was observed in the long-term dataset of Lam et al. [134], whereas the remaining studies reached values of approximately 1.05–1.3. This increase indicates a broadening of the molecular-weight distribution, consistent with the simultaneous presence of relatively high–molecular–weight chains and shorter products generated by random hydrolytic chain scission. The combined M_n_, M_w_, and PDI trends therefore indicate an initially limited molecular response followed by an increasingly heterogeneous and accelerated reduction in chain length during prolonged incubation. Dashed lines represent the fitted regression models and are intended to describe the overall kinetic trends rather than distinct mechanistic stages of PCL degradation.

### 3.2. Mass and Volume Loss

While molecular weight decay provides an internal chemical metric of macromolecular cleavage, gravimetric mass loss and structural volume reduction constitute the macroscopic manifestations of poly(ε-caprolactone) matrix dissolution. Because PCL undergoes bulk erosion under abiotic physiological conditions, these two physical parameters exhibit closely coupled, highly non-linear changes that govern the morphological and dimensional stability of the scaffold.

Consistent with the initial latency phase of intra-matrix chain scission, macroscopic mass loss remains functionally dormant until the bulk of the polymer transitions past its critical solubility threshold. In vitro studies consistently demonstrate that during the initial months of normothermic incubation (37 °C), the gravimetric decline of pristine PCL in simulated physiological fluids–such as PBS or SBF–is generally marginal [30].

Empirical profiling indicates that mass loss reaches a mere 0.5–0.7% after 8 weeks of immersion in PBS [100], and often hovers around a minor 0.9% even after a prolonged 24-week (approx. 6 months) timeline [148]. Long-term longitudinal analyses further underscore these sluggish kinetics; for instance, PCL discs immersed in PBS for 13 months exhibited an absolute gravimetric reduction of only 0.001 g [24].

This kinetic decoupling is governed by a classic bulk erosion mechanism, wherein water molecules rapidly infiltrate the entire volume of the polymer matrix, inducing random hydrolytic scission of the aliphatic ester bonds. Although this cleavage drives a continuous, progressive reduction in the M_n_, physical mass loss (Δm) only manifests when the macromolecular chains are cleaved down to a critical threshold—often reported in the range of 3000 to 5000 g/mol for specific experimental systems [91].

At this specific chemical juncture, the scission-generated short-chain oligomers and monomers achieve sufficient hydrophilicity to become water-soluble, enabling them to diffuse out of the hydrophobic bulk into the surrounding release medium. Consequently, complete bioresorption and total mass clearance of PCL under abiotic physiological environments is a long-term process, typically requiring between 2 and over 4 years [91]. However, it must be noted that terms such as “complete bioresorption” or “total dissolution” should be used cautiously when extrapolating solely from in vitro mass-loss measurements, as true biological resorption involves complex in vivo cellular and metabolic pathways. The accumulation of residual dry mass over time is evaluated via standard gravimetric profiling (4):

(4) $$\Delta m\;\left[\%\right]=\left(\frac{m_{0}-m_{t}}{m_{0}}\right)\cdot 100\%$$

where m_0_ represents the initial post-fabrication dry mass and mt denotes the dry mass at a designated temporal checkpoint t.

In contrast to surface-eroding polyesters that experience a linear decrease in external dimensions while maintaining bulk density, the volume loss (ΔV) of bulk-eroding PCL scaffolds exhibits unique spatial characteristics. During the early and middle stages of erosion, the external envelope (macroscopic volume) of the scaffold remains relatively static due to the structural template maintained by the lingering crystalline domains. Instead of immediate geometric shrinkage, the primary physical consequence of hydrolysis is an increase in internal micro-porosity and structural void volume. As soluble oligomers escape, dense polymer domains are systematically converted into interconnected, fluid-filled micro-channels, causing a severe drop in the true skeletal density (ρ_t_ = m_t_/V_t_). Only during the terminal phases of degradation, when the mechanical load-bearing capacity of these hollowed micro-struts is entirely compromised, does the scaffold undergo structural collapse, culminating in macroscopic geometric deformation and severe volumetric contraction. The specific pattern of volume change and structural collapse is strongly influenced by the initial scaffold geometry, porosity, degree of crystallinity, and internal architecture.

A critical experimental divergence occurs when evaluating mass loss profiles across different biomimetic media. PCL matrices consistently display lower apparent mass loss in SBF relative to PBS over identical temporal horizons [25]. This phenomenon does not signify a retardation of polymer hydrolysis, but rather a competitive gravimetric masking effect driven by surface mineralization dynamics. The underlying ester hydrolysis of the polymer backbone continues beneath the mineral layer and is not itself slowed by mineral deposition. The high ionic supersaturation of SBF induces the heterogeneous nucleation and growth of a carbonated hydroxyapatite layer on the polymer surface, exhibiting a calcium-to-phosphorus (Ca/P) molar ratio of approximately 1.65, which closely mimics natural bone mineralogy [29].

The continuous deposition of this dense, inorganic crystalline phase adds positive mass to the sample, mathematically offsetting and obscuring the simultaneous gravimetric loss occurring within the underlying organic polymer matrix. This is exemplified by reports indicating a 3.3% mass loss in PBS compared to a masked 2.3% loss in SBF over a 90-day period [25]. In bioinspired composites pre-loaded with inorganic nucleating agents (e.g., PCL-hydroxyapatite systems), this interface dynamic can completely dominate the gravimetric profile, yielding a net mass gain of up to 5.57% after just 14 days of SBF incubation due to intensive, accelerated biomimetic mineralization [149].

#### External and Structural Modulators of Erosion Kinetics

The baseline bulk erosion and gravimetric decline of PCL can be radically modulated or compressed through specific thermodynamic, biological, or structural vectors:

• Enzymatic Catalysis: In the presence of specific ester-cleaving enzymes such as lipase, PCL circumvents the slow bulk hydrolysis pathway entirely. The irreversible adsorption of enzymes to the solid–liquid interface triggers a rapid surface erosion pathway [28]. This direct, localized clipping cleaves surface chains directly into soluble monomers, driving near-total mass loss within days or weeks without requiring a prior, long-term drop in bulk Mn.
• Thermal Acceleration: Elevating the thermodynamic state of the system (e.g., to 47 °C) exponentially enhances the water diffusion coefficient and the reaction rate constant of ester hydrolysis in accordance with the Arrhenius law [150]. This forms the baseline for accelerated degradation protocols, compressing multi-year abiotic profiles into manageable laboratory timelines.
• Matrix Modification and Fillers: Blending PCL with highly hydrophilic polymers (such as PLGA) or integrating bioactive fillers (such as bioactive glass) severely disrupts the hydrophobic continuity of the PCL matrix [111,146]. These structural modifiers drastically increase thermodynamic water uptake (swelling ratio) and generate highly porous micro-interfaces that function as preferential fluid conduits, accelerating the rate of bulk hydrolysis and subsequent mass loss.

### 3.3. Mechanical Properties

**Figure 5:**
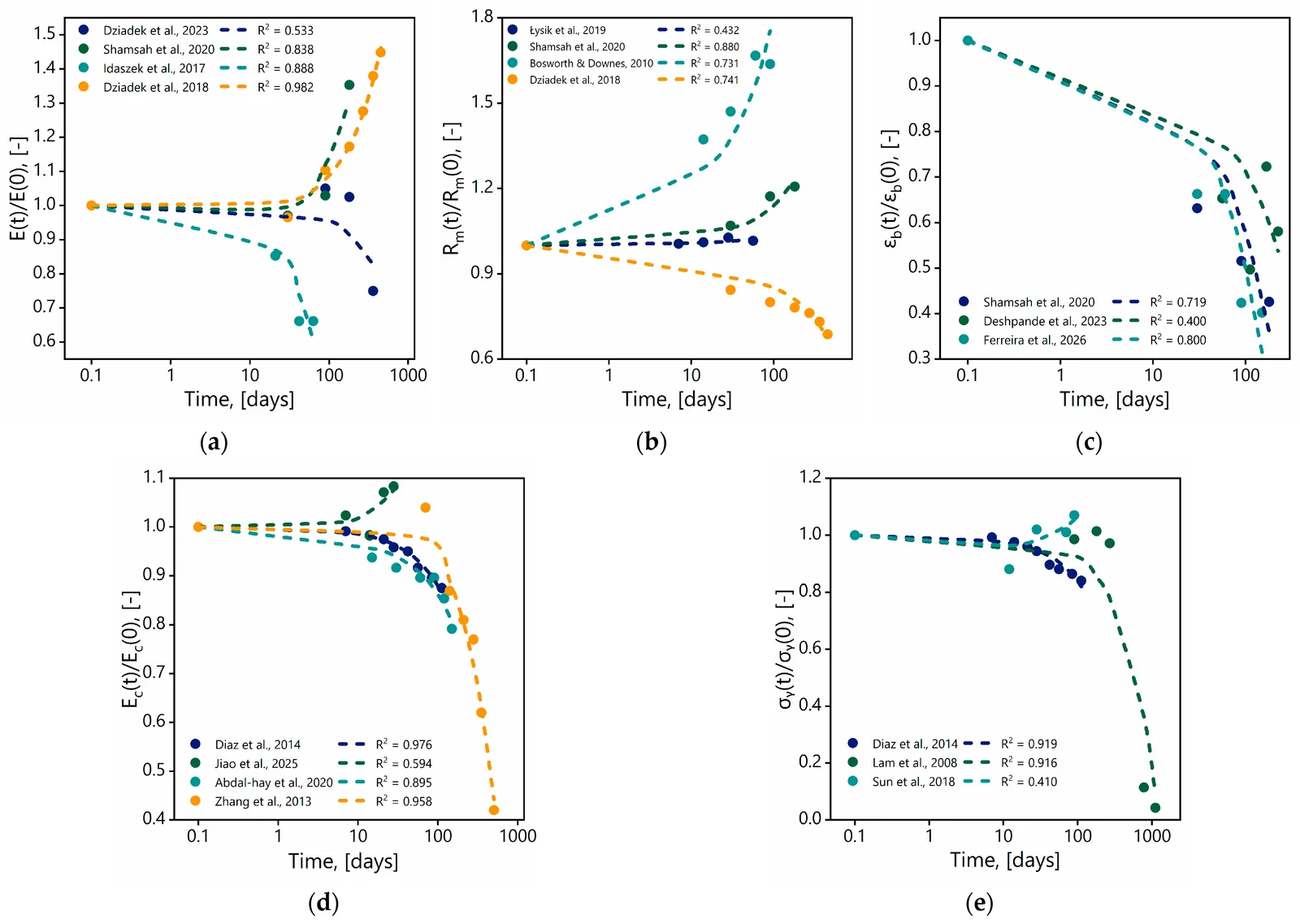
Kinetic evolution of PCL mechanical properties based on literature data. Data collected from studies conducted in SBF or PBS at 37 °C. (**a**) Normalized Young’s modulus (E), based on literature data [111,143,146,153]; (**b**) normalized tensile strength (R_m_). Data from the literature [100,143,151,153]; (**c**) normalized strain at break (ε_b_), based on literature data [26,143,154]; (**d**) normalized compressive modulus (E_c_), data from the literature [87,88,155,156]; and (**e**) normalized yield stress (σ_y_), Data from the literature [88,134,157]. Dashed lines represent the fitted linear regression model described by Equation (3) plotted against the logarithmic time scale.

The macroscopic consequences of PCL degradation are reflected in the progressive deterioration of its mechanical properties, resulting from chain scission, reduced molecular entanglement, mass loss, and changes in crystalline morphology [33,151]. These changes are commonly evaluated using quasi-static tensile and compression tests performed on universal testing machines. During tensile testing, Young’s modulus (E), yield strength (σ_y_), tensile strength (R_m_), and elongation at break (ε) are determined from the stress–strain response, whereas compression testing provides the compressive modulus and compressive strength. Strain can be measured using conventional extensometers or non-contact optical techniques, such as digital image correlation (DIC), which provide full-field measurements and enable the assessment of local strain heterogeneity [152]. To illustrate the mechanical evolution of PCL during degradation, literature-reported values of Young’s modulus, compressive modulus, yield strength, and tensile strength were compiled as a function of degradation time and are presented in Figure 5. Although direct comparison is limited by differences in specimen geometry, processing conditions, degradation media, and testing protocols, the collected data reveals general trends in the loss of stiffness, strength, and load-bearing capacity.

The mechanical performance of neat PCL during in vitro incubation under simulated physiological conditions exhibits substantial variability among the literature datasets, indicating that no single degradation trajectory can be assigned to all specimen architectures. The normalized tensile Young’s modulus, E(t)/E(0), shows both decreasing and increasing trends depending on the investigated material. Idaszek et al. [111] reported a relatively rapid reduction in tensile stiffness, with the modulus decreasing by approximately 25% during the first 50 days of immersion in SBF. A more pronounced long-term decline was observed by Dziadek et al. [146], particularly after approximately 100 days of incubation. By contrast, Shamsah et al. [143] and Dziadek et al. [153] reported an increase in normalized Young’s modulus, reaching approximately 1.3–1.4 times its initial value after about 100 days. Critically, these divergent responses are not contradictory but rather represent competing structural phenomena at the micro- and macroscopic levels. An initial increase in modulus is a classic mechanical signature of chemicrystallization; as the amorphous regions are hydrolytically cleaved, the shortened chains gain mobility and reorganize into highly ordered, rigid crystalline domains, thereby locally stiffening the matrix. Conversely, a macroscopic decrease in modulus occurs when the formation of internal voids, porosity, and mass loss physically outpaces this microscopic stiffening, ultimately compromising the load-bearing cross-section.

The normalized tensile strength, R_m_(t)/R_m_(0), also exhibited divergent behaviour. Łysik et al. [100] reported only minor changes, with the normalized values remaining close to unity throughout the investigated period. Shamsah et al. [143] observed a moderate increase at longer incubation times, while the data reported in [151] showed a substantially stronger rise. In contrast, the dataset reported in [153] indicated a progressive decrease in tensile strength during prolonged degradation. From a mechanistic perspective, these opposite trends indicate a transition in the internal load-bearing mechanism. A temporary rise in tensile strength reflects the increased crystallinity and rigidity of the degraded matrix. However, this is often a deceptive metric of structural health; as degradation progresses, the loss of molecular entanglement eventually overrides the crystalline reinforcement, leading to network deterioration and a subsequent drop in ultimate strength.

The normalized strain at break, εb(t)/εb(0), showed a pronounced decrease in all investigated systems, indicating a substantial loss of ductility during hydrolytic degradation. Although the rate of change differed among the datasets, the final normalized values decreased to approximately 0.4–0.6, corresponding to a 40–60% reduction relative to the initial strain at break. The strongest decline was observed in the datasets of Shamsah et al. [143] and Ferreira et al. [26], whereas Deshpande et al. [154] showed a slightly less pronounced but still substantial reduction. This universal, rapid decline makes strain at break the most sensitive and reliable macroscopic indicator of early-stage polymer degradation. Mechanistically, this phenomenon is driven by the preferential hydrolytic cleavage of amorphous “tie molecules”—the essential, flexible polymer chains that bridge and hold adjacent crystalline lamellae together. Once these inter-crystalline linkages are severed, the polymer matrix can no longer dissipate mechanical stress through plastic drawing or chain unfolding, resulting in severe embrittlement long before significant mass loss occurs.

The normalized compressive modulus, E_c_(t)/E_c_(0), generally decreased during incubation. Díaz et al. [88] and Abdal-hay et al. [155] reported gradual reductions in compressive stiffness, whereas the data from [156] showed a more pronounced long-term decline. In contrast, Jiao et al. [87] observed a slight increase during the investigated period. This short-term increase may reflect changes in the deformation behaviour of the porous architecture or structural rearrangement of the polymer phase. Nevertheless, the overall results indicate that prolonged hydrolysis generally reduces the resistance of PCL structures to compressive deformation, although the rate and magnitude of this reduction depend strongly on scaffold geometry and processing history.

The normalized yield stress, σ_y_(t)/σ_y_(0), showed the clearest deterioration during long-term degradation. Díaz et al. [88] reported a gradual reduction to approximately 0.83–0.85 of the initial value after about 100 days. Lam et al. [134] observed relatively small changes during the initial and intermediate stages, followed by a pronounced decline at extended incubation times. The normalized yield stress decreased to approximately 0.1 near 800 days and approached zero after approximately 1000 days, indicating an almost complete loss of resistance to irreversible deformation. Sun et al. [157] reported values fluctuating around the initial level, followed by a modest increase at longer times. However, the relatively low coefficient of determination for this regression indicates substantial scatter, and the apparent increase should therefore be interpreted cautiously.

In summary, these five mechanical parameters reveal a cascade of mechanical failure during PCL degradation. The process initiates with the rapid severing of amorphous tie chains, manifested macroscopically as a drastic loss of ductility (strain at break) and a transition toward brittle behavior. Simultaneously, chemicrystallization temporarily inflates the stiffness (Young’s modulus) and occasionally the tensile strength, masking the underlying chemical deterioration. Ultimately, as chain scission proceeds beyond the critical molecular weight threshold, macroscopic void formation and structural collapse occur, leading to the terminal deterioration of compressive and yield strengths. The preservation or temporary increase in stiffness during early incubation is therefore not indicative of structural stability, as it typically coexists with a substantial loss of ductility and fatigue resistance.

### 3.4. Thermal Properties

The degradation process of PCL is closely related to its thermal properties, which affect both the processing stability and the long-term performance of the material. Changes in molecular architecture induced by degradation may substantially affect the thermal transitions, crystallinity, and viscoelastic response of the polymer matrix [151,158]. Therefore, comprehensive thermal characterization is essential for understanding degradation mechanisms and their impact on the physicochemical properties of PCL. To this end, thermogravimetric analysis (TGA), differential scanning calorimetry (DSC), and dynamic mechanical analysis (DMA) are commonly employed to evaluate the thermal stability and temperature-dependent behavior of the material.

**Figure 6:**
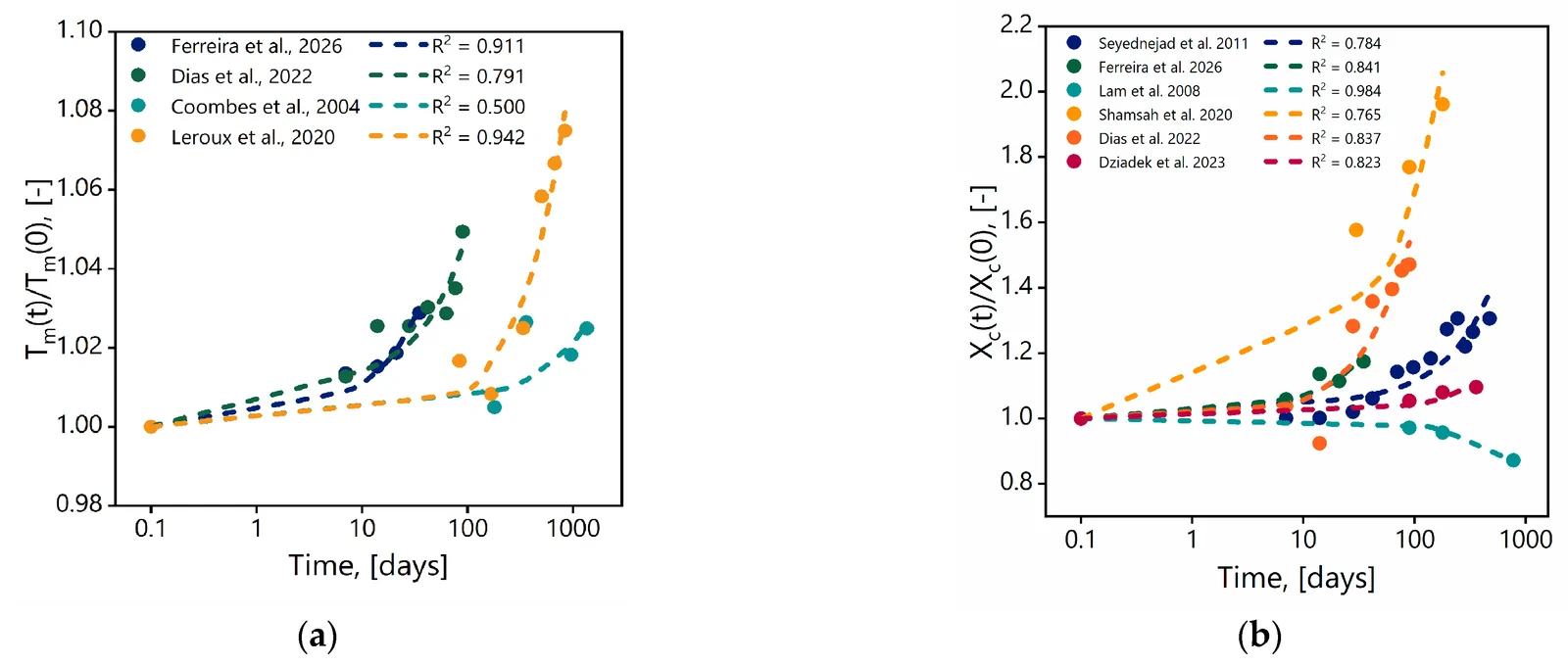
Kinetic evolution of PCL thermal and structural properties based on literature data. Data collected from studies conducted in SBF or PBS at 37 °C. (**a**) Normalized melting temperature (T_m_). Data from the literature [26,60,142,158]; and (**b**) normalized degree of crystallinity (X_c_). Data from the literature [26,30,60,134,143,146]. Dashed lines represent the fitted linear regression model described by Equation (3) plotted against the logarithmic time scale.

The TGA method was used to determine the thermal stability and decomposition profile of PCL by monitoring mass loss as a function of temperature [159]. The DSC study was used to evaluate thermal transitions, including melting and crystallization behavior, as well as degradation-induced changes in the degree of crystallinity [160]. The DMA analysis was used to investigate the temperature-dependent viscoelastic properties of the material, providing insight into structural changes within the polymer network during degradation [161]. To illustrate these phenomena, Figure 6 presents the kinetic evolution of PCL thermal and structural properties based on compiled literature data from studies conducted in SBF or PBS at 37 °C, specifically tracking changes in T_m_ and X_c_.

As shown in Figure 6, the thermal response of neat PCL during hydrolytic degradation is characterized by a relatively small increase in melting temperature and a substantially more variable evolution of crystallinity. The normalized melting temperature, T_m_(t)/T_m_(0), generally remained close to unity during the initial degradation period and increased gradually at longer incubation times. The magnitude of this increase differed among the investigated materials, ranging from only a few percent in the systems reported by Coombes et al. [142] to approximately 5–8% in the datasets of Dias et al. [60] and Leroux et al. [158] These results indicate that hydrolysis modifies the organization of the remaining polymer chains without producing a uniform thermal response across all PCL architectures.

The slight increase in T_m_ may be associated with preferential hydrolysis of less ordered amorphous regions and the subsequent enrichment of the residual material in more ordered crystalline domains. Hydrolytic chain scission can also increase the mobility of shorter polymer segments, allowing them to rearrange into more stable crystals. However, the normalized Tm values alone do not distinguish between recrystallization, crystal thickening, and the preferential removal of less ordered polymer fractions. Differences between data sets likely reflect the cumulative effects of initial molecular weight, crystallinity, processing history, sample geometry, and surface-to-volume ratio, rather than a single morphology-controlled mechanism.

More pronounced differences were observed in the evolution of the normalized degree of crystallinity, X_c_(t)/X_c_(0). Most datasets showed an increase in crystallinity during degradation, although both the onset and magnitude of this increase varied considerably. Shamsah et al. [143] reported the strongest response, with the normalized crystallinity approaching 2.0 after prolonged incubation. Dias et al. [60] observed an increase to approximately 1.5, whereas the datasets of Seyednejad et al. [30], Ferreira et al. [26], and Dziadek et al. [146] showed more moderate increases of approximately 10–30%. These trends are consistent with preferential degradation of amorphous regions and, in some cases, secondary crystallization of shorter and more mobile chain segments.

In contrast, Lam et al. [134] reported an approximately 9% decrease in the crystallinity of medical-grade PCL after 26 months of degradation in PBS, suggesting that prolonged hydrolysis progressively affected less perfect crystalline regions and crystal–amorphous interphases.

Overall, the compiled data demonstrates that changes in T_m_ and X_c_ are architecture- and time-dependent manifestations of hydrolytic chain scission and polymer reorganization. Standardized degradation conditions, specimen dimensions, and thermal-analysis procedures are therefore required to distinguish intrinsic changes in the PCL matrix from effects arising from processing-induced morphology and differences in mass transport.

## 4. In Vivo Degradation

Predicting the in vivo degradation kinetics of polycaprolactone necessitates an evaluation of the complex interplay between macromolecular architecture and the dynamic physiological environment, which often diverges significantly from controlled in vitro models. Upon implantation, PCL-based composites are subjected to reciprocal interactions with host inflammatory cascades, microvascular remodeling, cellular recruitment, and localized biomechanical loading [110]; a comprehensive summary of recent in vivo degradation studies evaluating these factors is systematically presented in Table 8.

To precisely align scaffold resorption with the temporal kinetics of tissue regeneration, molecular engineering strategies have focused on modulating polymer chain compositions. For instance, the synthesis of segmented PCL (SM–PCL) via aliphatic diisocyanates facilitates highly programmable resorption pathways [162]. In a twelve-month murine model, thin, porous films of SM–PCL underwent near-complete resorption within six months, whereas dense matrices exhibited an 82% reduction in molecular weight. Conversely, homopolymer PCL retained 47% of its initial M_w_ over the identical period, confirming that structural segmentation directly dictates the hydrolytic profile [162].

Furthermore, the choice of polymer grade remains a critical determinant of mechanical stability; medical-grade PCL implemented in 3D-printed tracheal scaffolds demonstrated accelerated degradation compared to its research-grade counterpart, exhibiting a 17.5% reduction in M_w_ and concomitant mechanical weakening over six months under load-bearing conditions [163]. Beyond macromolecular chemistry, macroscopic geometry and surface-area-to-volume ratios fundamentally govern the fluid-biomaterial interface.

Long-term clinical evaluations of PCL-based dermal fillers indicate a biphasic degradation mechanism, characterized by an initial isovolemic phase lasting up to three years wherein M_w_ decreases without volumetric loss, followed by catastrophic bulk degradation and rapid mass transport in the fourth year [103]. Maximizing the exposed surface area can further refine these kinetics; porous PCL microspheres undergo a more gradual and predictable hydrolytic erosion relative to solid particulate systems, while simultaneously driving the up-regulated expression of types I and III collagen to enhance regenerative outcomes [22].

To broaden the functional window of PCL, composite formulation and blending are widely deployed. Blending PCL with sebacic acid accelerates hydrolytic cleavage, yielding injectable matrices tailored for bone regeneration that circumvent chronic inflammatory responses [164]. Leveraging amphiphilic copolymers, PCL/Pluronic F68 implants have achieved near–zero–order release kinetics of therapeutic payloads, such as mifepristone, sustained over 180 days for the suppression of endometriosis [165]. Within the context of hard-tissue engineering, blending distinct molecular weight fractions of PCL and incorporating osteoinductive agents like simvastatin allows investigators to precisely synchronize scaffold degradation with the rate of de novo bone apposition [166].

A critical consideration in long-term load-bearing applications is the non-linear decoupling of mechanical integrity from total mass loss. Under physiological conditions, 3D-printed PCL plates can experience a ~70% reduction in tensile strength after 24 weeks despite exhibiting less than 1% total mass loss [167]. This accelerated loss of structural competence correlates with rapid in vivo chain scission, with significant M_w_ reductions manifest within 12 weeks. Indeed, comparative analyses confirm that in vivo environments accelerate M_w_ loss (14%) relative to in vitro controls (7%) over identical intervals, even when macro-structural integrity is preserved for up to six months [91].

For critical-sized cranial or segmental defects where immediate mechanical performance is mandatory, compounding PCL with bioactive ceramics, such as biphasic calcium phosphate (BCP) or hydroxyapatite, effectively mitigates early structural failure, preserving necessary load-bearing capacity while maintaining a controlled degradation profile compatible with osseous remodeling [144,168].

**Table 8:** Summary degradation in vivo of PCL.

Composition	Structure Type	Preparation Method	Implantation Site	Time	Observations	Reference
Polycaprolactone (medical-grade and research-grade)	Patient-specific, segmental tracheal scaffolds	Extrusion-based 3D printing	Tracheal defects of rabbits (*n* = 8)	6 months	Medical-grade PCL scaffolds showed higher strength, strain, and better tissue regeneration than research-grade PCL. However, they degraded faster in the body, with a noticeable decrease in molecular weight and mechanical strength after transplantation.	[163]
Segmented–polycaprolactone	Solid and porous, thin films	Extraction methods	Left thigh muscle of male rats (*n* = unknown)	12 months	SM–PCL materials degraded faster than pure PCL, showing a greater decrease in molecular weight during implantation and was completely degraded 6 months after implantation. Despite degradation, no significant pathological reactions were observed, indicating good biocompatibility and tissue compatibility.	[162]
DMFI300: 30 wt% porous PCL microspheres in a 70 wt% Na-CMC gel carrier	Injectable porous microsphere filler	Commercial filler supplied in a prefilled syringe	Dorsal region of rabbits (*n* = 10) and hairless mice (*n* = 5 per group)	30 months for rabbits and 12 weeks for mice	The porous PCL microspheres gradually biodegraded in vivo, with most particles disappearing by 24 months and complete degradation observed by 30 months after injection. The material showed good tissue safety, inducing only mild inflammatory response while stimulating collagen production around the microspheres.	[27]
Polycaprolactone (PCL-M2)	PCL microspheres suspended in a CMC-based injectable gel	Commercial injectable dermal filler	Injection below the hairline on the face in humans (*n* = 8)	4 years	Particles exhibited isovolemic degradation, maintaining constant size and volume for the first 3 years (Phase-1). Significant size reduction occurred only in the 4th year due to the onset of bulk degradation (Phase-2).	[103]
PCL + Sebacic Acid (SA)	Injectable, in situ-crosslinking gel	Solution blending	Subcutaneous and femoral bone of rats (*n* = 15)	90 days	The PCL-SA gel blend is biocompatible and promotes tissue regeneration in both subcutaneous and bone environments. It exhibits an adequate degradation rate suitable for bone tissue engineering applications	[164]
PCL, Pluronic F68, and Mifepristone	Hollow cylindrical capsule implant	Solution blending	Dorsal subcutaneous region female rats (*n* = 84)	6 months	Subcutaneous implantation of mifepristone-loaded capsules effectively treated endometriosis in rats through sustained, zero-order drug release. The treatment showed a significant dose-dependent inhibitory effect on endometrial explant growth.	[165]
Polycaprolactone and Simvastatin	Spiral-wound, nanofibrous 3D scaffolds	Electrospinning	Cranial bone defects of rats (*n* = 90)	6 months	Simvastatin-loaded 3D scaffolds promoted significant bone mineralization and osseous integration in cranial defects. The best results were achieved by incorporating the drug directly during the electrospinning process.	[166]
PCL/β-TCP (80/20, *w*/*w*)	Porous cranioplasty plates	Fused deposition modelling (FDM)	Cranial bone defects of female rabbits (*n* = unknown)	12 weeks	Molecular weight decreased by 9.67% in vivo after 12 weeks. While mass loss was minimal (0.79%), tensile strength dropped significantly by 69.30% under simulated physiological conditions.	[167]
PCL/BCP (80/20, *w*/*w*) and PCL/β-TCP (80/20, *w*/*w*)	Cylindrical porous scaffolds	Melt Stretching and Compression Molding (MSCM); 3D-printing (FDM)	Subcutaneous (dorsal) of male rats (*n* = 12)	90 days	Scaffolds were highly biocompatible, showing neo-vascularization and collagen formation throughout the pores. Significant volume loss (~30%) was observed, though molecular weight changes remained slight during the study period.	[168]
PCL/β-TCP (80/20, *w*/*w*)	Porous 3D scaffolds	3D-printing (FDM)	Subcutaneous (SC) and Intramuscular (IM) critical-size defects in the calvarium of rabbits (*n* = unknown)	2 years	In vivo mass loss was low, reaching 7% for PCL-β-TCP composites and only 1% for pure PCL scaffolds.	[91]
Medical-grade PCL/HA (96/4, *w*/*w*)	3D-printed porous scaffolds with a Voronoi design	3D-printing (FDM)	Ectopic (subcutaneous) of male rats (*n* = 2)	8 weeks	The Voronoi design supported high vascularization without extensive fibrous encapsulation.	[144]
PCL	Dense membrane	Solvent casting followed by ethylene oxide sterilization	Pleural cavity of Landrace pigs; animals were assigned to PCL-membrane and mechanical-abrasion groups (total *n* = 24)	12 months	Macroscopic analysis showed uniform pleural adhesion with greater counter-peel strength than mechanical abrasion at 6 and 12 months. The PCL membrane exhibited gradual biodegradation, losing 46.7% of its molecular weight by month 12. Histopathology revealed sustained angiogenesis and increased α-actin expression, supporting homogeneous tissue repair without mortality, systemic toxicity, genotoxicity, or carcinogenicity.	[169]

### The Translational Gap: Contrasting In Vitro and In Vivo Degradation Profiles

A critical challenge in the design of PCL-based biomedical devices is the discrepancy that may arise between degradation behavior observed under simplified in vitro conditions and that occurring after implantation. Although in vitro models employing abiotic media such as PBS or SBF are essential for investigating fundamental hydrolytic mechanisms, they do not fully reproduce the biochemical, cellular, fluidic, and mechanical complexity of the physiological environment. Therefore, degradation kinetics obtained under simplified in vitro conditions cannot be directly extrapolated to predict the clinical lifetime of PCL-based devices. The degradation rate and mechanism may differ substantially depending on polymer molecular weight, crystallinity, construct geometry, implantation site, and biological environment [33,60].

In vitro degradation of PCL is predominantly associated with hydrolytic cleavage of ester bonds, whereas the physiological environment introduces additional factors, including enzymatic activity, cellular interactions, inflammatory responses, and phagocytic processes. Macrophages and foreign-body giant cells have been observed around implanted PCL materials and may contribute to the biological processing and resorption of polymer fragments [33,170].

The importance of these biological factors is illustrated by direct comparisons of in vitro and in vivo degradation. For example, Lam et al. [91] reported limited molecular-weight and mass changes of PCL-based scaffolds after six months in both PBS and a rabbit implantation model, whereas other studies have demonstrated substantially different degradation kinetics depending on scaffold architecture and enzymatic conditions. In particular, How et al. [169] observed faster degradation of electrospun PCL under selected in vitro enzymatic conditions than in vivo, highlighting that the relationship between in vitro and in vivo degradation cannot be generalized.

The long-term persistence of PCL in vivo further emphasizes the importance of considering the specific material and implantation conditions. Sun et al. [51] reported that PCL implants with an initial molecular weight of approximately 66 kDa retained their macroscopic shape for up to two years in rats, with fragmentation into lower-molecular-weight pieces observed after approximately 30 months.

Consequently, the translation of degradation data from in vitro models to physiological performance should be considered system-specific rather than based on a universal in vivo acceleration factor. Anatomical location, vascularization, mechanical loading, scaffold architecture, molecular weight, crystallinity, enzymatic exposure, and the host inflammatory response can all influence the degradation trajectory.

## 5. Conclusions and Future Perspectives

This study comprehensively evaluated the degradation behavior of PCL in the context of its intrinsic properties, environmental conditions, and material modifications. The compiled literature data indicate the baseline degradation of neat PCL under standard in vitro conditions progresses via a non-enzymatic, random chain scission hydrolytic mechanism, which is primarily governed by bulk water uptake. Due to its high crystallinity and inherent hydrophobicity, this process features remarkably slow kinetics, guaranteeing long-term structural stability but simultaneously limiting its suitability for fast-paced tissue regeneration.

To modulate these degradation kinetics, the incorporation of secondary polymer phases and bioceramics represents a crucial milestone. The success of these modifications, however, depends on the specific polymer grade, initial porosity, composite formulation, and experimental conditions used. Under appropriate parameters, the addition of bioceramics introduces a vital buffering effect that localizes and neutralizes acidic degradation byproducts while shifting the mechanism from pure mass loss to a dynamic ion-exchange process that promotes surface mineralization.

Furthermore, in contrast to purely hydrolytic aging, exposure to specific enzymatic environments, such as lipase-mediated degradation, significantly accelerates PCL mass loss by operating predominantly through a surface erosion mechanism, effectively bypassing the slow bulk hydrolysis and demonstrating that the functional lifetime of PCL constructs can be finely tuned via biological or biochemical cues.

Despite the extensive body of literature on aliphatic polyesters, several critical variables driving PCL degradation kinetics and structural performance during long-term incubation remain poorly characterized, highlighting vital directions for future research. A significant limitation in current in vitro aging models is the ambiguity surrounding medium exchange protocols, where static or inadequately replenished setups lead to the local accumulation of degraded low-molecular-weight fragments that cause artificial autocatalysis; therefore, future studies must systematically contrast static versus dynamic degradation environments to decouple the effects of media replenishment from intrinsic polymer chemistry. Furthermore, future assessments must rigorously maintain the distinction between molecular weight reduction (chain scission), macroscopic mass loss (erosion), and actual physiological bioresorption (metabolic clearance), as these parameters do not occur simultaneously and physical disintegration does not equate to total biological elimination.

Additionally, while the baseline processing of neat PCL is well-understood due to its favorable thermal stability and low melting point, the introduction of particulate bioceramics or secondary polymer phases substantially alters the melting behavior. To transition towards predictable advanced biofabrication via technologies such as Fused Deposition Modeling or electrospinning, comprehensive rheological characterization focusing on melt viscosity, viscoelastic moduli, and shear-thinning profiles is strictly required to prevent nozzle clogging and ensure structural fidelity. Most degradation studies focus primarily on mass loss and molecular weight reduction, frequently neglecting the evolution of mechanical competence over time. Because the deterioration of molecular weight inevitably precedes macroscopic structural failure, monitoring changes in compressive strength and stiffness in vitro as a function of degradation time is critical to ensure that the composite scaffold sustains physiological loads until it is progressively replaced by neo-formed bone tissue.

Future research should focus on the design and synthesis of novel multi-component systems leveraging the polycaprolactone matrix; incorporating bioactive secondary phases such as functionalized bioceramics or bioglasses could help overcome the intrinsic limitations of hydrophobicity and biological inertness. Developing these polymer-ceramic formulations would tailor degradation kinetics to match bone ingrowth, promote mineralization via hydroxyapatite formation, and enhance the osteoinductive potential of the constructs for bone tissue engineering applications.

## Figures and Tables

**Table 1:** A list of PCL’s mechanical and physical characteristics [8,9].

Properties	Values
Number-average molecular weight M_n_, g/mol	3000 to 80,000
Density, g/cm^3^	1.07 to 1.20
Melting temperature (T_m_), °C	56 to 64
Glass transition temp. (T_g_), °C	−65 to −60
Degradation time, years	2 to 4
Tensile strength (R_m_), MPa	4 to 24
Young modulus (E), MPa	210 to 440
Degree of Crystallinity (X_c_), %	45 to 70

**Table 2:** Effect of Polymer Parameters on PCL Degradation Rate.

Parameter	Crystallinity	Molecular Weight	Polydispersity Index
Change	Increase (↑)	Increase (↑)	Increase (↑)
Effect on Degradation Rate	Slower (↓)	Slower (↓)	Generally slower (↓)
Underlying Mechanism	Highly ordered crystalline regions act as physical barriers restricting the diffusion of water and enzymes into the polymer matrix.	Longer polymer chains contain fewer chain ends and require more cleavage events to break down into soluble water fragments.	A broad molecular weight distribution containing very long chains can increase structural integrity and prolong the time required for complete mass loss.

**Table 5:** Influence of PCL Geometry and Morphology on Degradation Mechanics and Kinetics.

Property/Geometric Form	Bulk and Monolithic Forms	Thin Polymeric Films	3D Porous Scaffolds	Micro- and Nanofibers	Micro- and Nanofibers/Particles
Primary Degradation Mechanism	Heterogeneous bulk erosion	Surface-associated erosion or homogeneous bulk erosion	Porosity-dependent; balance between surface and bulk erosion	Rapid surface-associated erosion	Accelerated, pure surface erosion
Mass Loss Rate	Extremely slow; negligible during initial phases	Moderate; strongly mediated by thickness and crystallinity	Governed by SA/V ratio; accelerated via accessible porosity	Rapid (particularly for sub-micron diameters)	Extremely rapid mass loss profile
Autocatalytic Intensity	High; pronounced accumulation of acidic byproducts in the core	Low; short diffusion pathways prevent oligomer entrapment	Mitigated; interconnected pores facilitate byproduct flushing	Minimal; maximal surface exposure to surrounding media	Absent; instantaneous diffusion of oligomers into the buffer
Critical Geometric Parameter	Cross-sectional thickness	Surface topography and spherulitic morphology	Porosity, pore interconnectivity, and local SA/V ratio	Fiber diameter and drawing/stretching ratio	Specific surface area-to-volume ratio
Molecular Weight Evolution	Catastrophic drop in the inner core (skin-core model)	Homogeneous and uniform decrease throughout the volume	Slower M_n_ drop compared to bulk forms due to pore perfusion	Highly dependent on processing-induced chain orientation	Immediate and uniform chain fragmentation

**Table 6:** Criteria for selecting a degradation medium for PCL [109,110].

Feature	PBS	SBF
Primary use	Pure hydrolytic degradation	Bioactivity and Mineralization
Material goal	General scaffold stability	Bone-contacting applications
Degradation	Slow, uniform (surface)	Complex (degradation + mineral deposition)
Mineralization	None (or minimal)	High (apatite formation)
Recommended for	Long-term in vitro studies	Early, middle, and long-term surface studies

## Data Availability

No new experimental data were generated in this study. The data analyzed and discussed in this review were obtained from previously published studies, all of which are cited in the manuscript. Data sharing is not applicable to this article.

## Supplementary Materials

The following supporting information can be downloaded at: https://www.mdpi.com/article/10.3390/ma19183894/s1. The supplementary file contains detailed parameters obtained from the mathematical models fitted to the degradation data from individual studies, including model coefficients and R^2^ values.

## Abbreviations

The following abbreviations are used in this manuscript:

3D	Three-Dimensional
6-HCA	6-Hydroxycaproic Acid
β-TCP	Beta-Tricalcium Phosphate
BG	Bioactive Glass
CALB	Candida Antarctica Lipase B
DSC	Differential Scanning Calorimetry
E	Young’s Modulus
Ec	Compressive Modulus
εb	Strain at Break
GO	Graphene Oxide
HA	Hydroxyapatite
nHA	Nano-Hydroxyapatite
M_n_	Number-Average Molecular Weight
M_w_	Weight-Average Molecular Weight
PBS	Phosphate-Buffered Saline
PCL	Poly(ε-caprolactone)
PDI	Polydispersity Index
PEG	Poly(ethylene glycol)
PGA	Poly(glycolic acid)
PHAs	Polyhydroxyalkanoates
PLA	Poly(lactic acid)
PLGA	Poly(lactic-co-glycolic acid)
PLLA	Poly(L-lactic acid)
R_m_	Ultimate Tensile Strength
SA/V	Surface Area-to-Volume Ratio
SBF	Simulated Body Fluid
SEM	Scanning Electron Microscopy
σ_y_	Yield Strength
T_g_	Glass Transition Temperature
T_m_	Melting Temperature
X_c_	Degree of Crystallinity
