# Supplementary material for: Understanding Polycaprolactone Degradation: Molecular Mechanisms and Implications for Biomedical Device Design
Source: Materials (Basel). 2026 Sep 12;19(18):3894. doi: 10.3390/ma19183894 (PMC13608729; doi:10.3390/ma19183894)
Supplement: Supplementary file 1 [file materials-19-03894-s001.zip › materials-4512357-supplementary.pdf]

Analyzed Property Mn; Exponential Model

Reference	Kinetic Constant λ'±SE	R^2
Seyednejad, 2011	$7.60\cdot10^{-4}\pm4.15\cdot10^{-5}$	0.968
Houglund, 2007	$1.24\cdot10^{-3}\pm1.36\cdot10^{-4}$	0.908
Houglund, 2007	$5.04\cdot10^{-3}\pm6.37\cdot10^{-4}$	0.947
Shamsah, 2020	$3.11\cdot10^{-3}\pm4.57\cdot10^{-4}$	0.876
Laubach, 2023	$9.62\cdot10^{-4}\pm4.67\cdot10^{-5}$	0.966
Coombes, 2003	$4.11\cdot10^{-4}\pm3.93\cdot10^{-5}$	0.943

Analyzed Property Mw; Exponential Model

Reference	Kinetic Constant λ'±SE	R^2
Poh, 2016	$3.05\cdot10^{-4}\pm1.74\cdot10^{-5}$	0.946
Park, 2010	$2.15\cdot10^{-3}\pm8.59\cdot10^{-5}$	0.989
Kim,2025	$2.79\cdot10^{-3}\pm1.11\cdot10^{-4}$	0.995
Shamsah, 2020	$1.29\cdot10^{-3}\pm3.20\cdot10^{-4}$	0.951
Dziadek, 2023	$8.81\cdot10^{-4}\pm4.67\cdot10^{-5}$	0.993
Coombes, 2003	$2.23\cdot10^{-4}\pm4.29\cdot10^{-6}$	0.997

Analyzed Property PDI; Linear Model

Reference	Slope a ±SE	Intercept b ±SE	R^2
Park, 2010	$3.35\cdot10^{-4}\pm1.67\cdot10^{-5}$	$1.00\pm9.37\cdot10^{-4}$	0.995
Houglund, 2007	$1.94\cdot10^{-4}\pm1.47\cdot10^{-5}$	$0.99\pm2.33\cdot10^{-4}$	0.972
Houglund, 2007	$3.99\cdot10^{-4}\pm7.83\cdot10^{-7}$	$1.00\pm1.24\cdot10^{-4}$	1.000
Shamsah, 2020	$1.29\cdot10^{-3}\pm7.80\cdot10^{-5}$	$1.01\pm1.29\cdot10^{-3}$	0.993
Coombes, 2003	$2.21\cdot10^{-4}\pm6.19\cdot10^{-6}$	$0.98\pm3.74\cdot10^{-6}$	0.995
Lam, 2008	$5.12\cdot10^{-4}\pm1.55\cdot10^{-5}$	$1.01\pm6.22\cdot10^{-3}$	0.998

Analyzed Property E; Linear Model

Reference	Slope a ±SE	Intercept b ±SE	R^2
Dziadek, 2023	$-4.76\cdot10^{-2}\pm1.67\cdot10^{-5}$	1.00	0.533
Shamsah, 2020	$2.06\cdot10^{-3}\pm6.38\cdot10^{-4}$	$0.93\pm6.49\cdot10^{-2}$	0.838
Idaszek, 2017	$-6.25\cdot10^{-3}\pm7.00\cdot10^{-4}$	1.00	0.888
Dziadek, 2018	$1.07\cdot10^{-3}\pm6.53\cdot10^{-5}$	$0.98\pm1.64\cdot10^{-2}$	0.982

Analyzed Property R_m; Linear Model

Reference	Slope a ±SE	Intercept b ±SE	R^2
Łysik, 2019	$3.09\cdot10^{-4}\pm2.05\cdot10^{-4}$	$1.01\pm5.91\cdot10^{-3}$	0.432
Shamsah, 2020	$1.12\cdot10^{-3}\pm2.93\cdot10^{-4}$	$1.03\pm2.93\cdot10^{-2}$	0.880
Bosworth, 2010	$6.34\cdot10^{-3}\pm7.00\cdot10^{-4}$	1.18 ± 0.11	0.731
Dziadek, 2013	$-5.09\cdot10^{-4}\pm1.34\cdot10^{-4}$	$0.90\pm3.40\cdot10^{-2}$	0.741

Analyzed Property ε_b; Linear Model

Reference	Slope a ±SE	Intercept b ±SE	R^2
Shamsah, 2020	$-2.69 \cdot 10^{-3} \pm 1.19 \cdot 10^{-3}$	0.85±0.12	0.719
Deshpande, 2020	$-1.37 \cdot 10^{-3} \pm 9.71 \cdot 10^{-4}$	0.85±0.13	0.400
Ferreira, 2026	$-3.75 \cdot 10^{-3} \pm 1.08 \cdot 10^{-3}$	0.88±0.09	0.800

Analyzed Property E_c; Linear Model

Reference	Slope a ±SE	Intercept b ±SE	R^2
Diaz, 2014	$-1.17 \cdot 10^{-3} \pm 6.95 \cdot 10^{-5}$	$1.00 \pm 3.74 \cdot 10^{-3}$	0.976
Jiao, 2025	$3.07 \cdot 10^{-3} \pm 1.47 \cdot 10^{-3}$	0.99±0.03	0.594
Abdal-hay, 2020	$-1.10 \cdot 10^{-3} \pm 1.69 \cdot 10^{-4}$	0.97±0.01	0.895
Zhang, 2013	$-1.23 \cdot 10^{-3} \pm 1.15 \cdot 10^{-4}$	1.06±0.3	0.958

Analyzed Property σ_y; Linear Model

Reference	Slope a ±SE	Intercept b ±SE	R^2
Diaz, 2014	$-1.51 \cdot 10^{-3} \pm 1.70 \cdot 10^{-4}$	$0.99 \pm 9.12 \cdot 10^{-3}$	0.919
Lam, 2008	$-8.18 \cdot 10^{-4} \pm 7.98 \cdot 10^{-5}$	1.00	0.904
Sun, 2018	$1.17 \cdot 10^{-3} \pm 8.09 \cdot 10^{-4}$	0.95±0.04	0.410

Analyzed Property T_m; Linear Model

Reference	Slope a ±SE	Intercept b ±SE	R^2
Ferreira, 2026	$7.38 \cdot 10^{-4} \pm 1.33 \cdot 10^{-4}$	$1.00 \pm 2.61 \cdot 10^{-3}$	0.911
Dias, 2022	$3.89 \cdot 10^{-4} \pm 8.16 \cdot 10^{-5}$	$1.01 \pm 4.16 \cdot 10^{-3}$	0.791
Coombes, 2003	$1.48 \cdot 10^{-6} \pm 8.58 \cdot 10^{-6}$	$1.01 \pm 6.54 \cdot 10\text{-}3$	0.500
Leroux, 2020	$9.38 \cdot 10^{-5} \pm 1.04 \cdot 10^{-5}$	$1.00 \pm 4.91 \cdot 10^{-3}$	0.942

Analyzed Property X_c; Linear Model

Reference	Slope a ±SE	Intercept b ±SE	R^2
Seyednejad, 2011	$7.19 \cdot 10^{-4} \pm 1.13 \cdot 10^{-4}$	1.04±0.02	0.784
Ferreira, 2026	$4.68 \cdot 10^{-3} \pm 1.17 \cdot 10^{-3}$	1.02±0.02	0.841
Lam, 2008	$-1.56 \cdot 10^{-4} \pm 1.42 \cdot 10^{-5}$	$0.99 \pm 5.70 \cdot 10^{-3}$	0.984
Shamsah, 2020	$4.58 \cdot 10^{-3} \pm 1.80 \cdot 10^{-3}$	1.23±0.18	0.765
Dias, 2022	$5.98 \cdot 10^{-3} \pm 1.08 \cdot 10^{-3}$	1.00±0.06	0.837
Dziadek, 2023	$2.48 \cdot 10^{-4} \pm 8.14 \cdot 10^{-5}$	1.02±0.02	0.823
